# Temporal shifts in antiretroviral therapy regimens and metabolic outcomes in people living with HIV: A systematic review and meta‐analysis

**DOI:** 10.1111/hiv.70260

**Published:** 2026-05-21

**Authors:** Melani Ratih Mahanani, Myo Chit, Sophie Mohr, Elisenda Cama i Gibernau, Svetlana Hetjens, Volker Winkler, Hans‐Michael Steffen, Florian Neuhann

**Affiliations:** ^1^ Heidelberg Institute of Global Health, Heidelberg University Hospital Heidelberg Germany; ^2^ Division of Prevention of Cardiovascular and Metabolic Diseases, Medical Faculty Mannheim, Centre for Preventive Medicine and Digital Health Heidelberg University Mannheim Germany; ^3^ Department of Medical Statistics and Biomathematics, Medical Faculty Mannheim Heidelberg University Mannheim Germany; ^4^ Department of Gastroenterology and Hepatology Faculty of Medicine and University Hospital Cologne, University of Cologne Cologne Germany; ^5^ Faculty of Medicine and University Hospital Cologne, Hypertension Centre, University of Cologne Cologne Germany; ^6^ Department of Postgraduate Studies and Research Chreso University Lusaka Zambia; ^7^ School of Medicine and Clinical Sciences Levy Mwanawasa Medical University Lusaka Zambia

**Keywords:** anti‐HIV agents, HIV, metabolic outcome, metabolic syndrome, type 2 diabetes mellitus

## Abstract

**Introduction:**

Antiretroviral therapy (ART) for people living with HIV has led to dramatically reduced mortality and improved life expectancy. This achievement is accompanied by a higher risk for metabolic and other non‐communicable diseases. The role and contribution of various ART regimens to adverse metabolic outcomes are not fully understood. We aimed to systematically evaluate the risk of metabolic outcomes associated with ART and to describe temporal trends in these risks across the modern ART era.

**Methods:**

Following our prespecified, registered protocol, we conducted a systematic review and meta‐analysis of non‐randomized observational studies, comparing ART‐treated and ART‐naïve adult people living with HIV and report the metabolic outcomes of interest. We extracted ART regimen combinations and metabolic outcomes and calculated pooled odds ratios (ORs) with 95% confidence intervals (95% CI) using a random‐effects model. Temporal patterns were summarized by publication year and year of ART rollout.

**Results:**

We identified 6827 studies, in which 39 studies were eligible for analysis with information from 24 632 people living with HIV. Nucleoside reverse transcriptase inhibitors (NRTIs) were present throughout the review period. Non‐nucleoside reverse transcriptase inhibitors (NNRTIs) predominated from the early 2000s to 2014, while protease inhibitors (PIs) dominated 1998–2014. Integrase strand transfer inhibitors (INSTIs) appeared from 2016 onwards. ART‐treated adult people living with HIV had higher odds for metabolic syndrome (OR = 2.16, 95% CI = 1.33–3.52), while no statistically significant association was observed for type 2 diabetes mellitus (T2DM) (OR = 1.37, 95% CI = 0.65–2.90).

**Discussion:**

Metabolic burden associated with modern HIV treatment shifted from lipid abnormalities to weight‐ and glucose‐related outcomes. Meta‐analysis results showed that ART‐treated adults had nearly twice the odds of metabolic syndrome compared with ART‐naïve adults. In contrast, our meta‐analytic evidence did not demonstrate a statistically significant association between ART use and the odds of developing T2DM. Findings should be interpreted with caution given potential confounding by disease stage, access to healthcare and survivorship bias.

**Conclusions:**

ART regimens and their metabolic outcomes have shifted over time. ART‐treated adult people living with HIV had higher odds of metabolic syndrome than their ART‐naïve peers. Given heterogeneity and residual confounding, robust, long‐term studies would be needed to refine regimen‐specific risks.

## INTRODUCTION

The introduction of antiretroviral therapy (ART) has transformed Human Immunodeficiency Virus (HIV) infection from a fatal disease to a manageable chronic condition, dramatically reducing mortality and improving life expectancy in people living with HIV [1, 2]. The evolution of antiretroviral treatment over the past decades has seen significant advancements in efficacy, safety and convenience, leading to decreased pill burden and improved treatment adherence [3, 4].

Modern ART typically consists of a combination of three drugs from different antiretroviral classes. The principal classes include nucleoside reverse transcriptase inhibitors (NRTIs), non‐nucleoside reverse transcriptase inhibitors (NNRTIs), protease inhibitors (PIs) and integrase strand transfer inhibitors (INSTIs) [5]. The introduction of these combinations has led to sustained viral suppression, immune reconstitution and prevention of opportunistic infections, transforming the clinical course of HIV infection [6].

While ART has dramatically reduced HIV‐related mortality, people living with HIV receiving ART now have longer life expectancy, which in turn increases their risk of developing chronic non‐communicable diseases (NCDs) due to factors like aging, lifestyle, chronic inflammation and potential side effects of long‐term ART exposure [7]. Particularly, the growing prevalence of overweight and obesity among people living with HIV further complicates the clinical picture, potentially exacerbating ART‐associated metabolic abnormalities [8].

Although previous studies have examined specific associations between ART and metabolic outcomes, comprehensive analyses of differential effects across ART classes and individual agents are limited. Previous studies [9, 10, 11] reviewed metabolic outcomes affecting adipose tissue, lipid and glucose metabolism in people living with HIV on ART but did not extensively compare different ART classes or focused on only one ART class. Wenjing et al. [12] provided an important synthesis showing high dyslipidaemia prevalence in people living with HIV and signalled that INSTI‐based regimens are associated with lower HDL‐C. Their scope, however, was limited to lipid abnormalities and a single country context. A network meta‐analysis of randomized trials compared first‐line ART regimens but did not assess longer‐term outcomes such as metabolic outcomes [13]. To our knowledge, integrated global analyses of multiple metabolic outcomes across different ART regimens have not been studied before.

By examining the relationships between specific ART regimens and metabolic outcomes, our systematic literature review and meta‐analysis aim to assess which ART impacts NCDs and to describe temporal trends in regimen use and reported outcomes across the modern ART era.

## METHODS

### Protocol registration

This systematic review and meta‐analysis were conducted according to a prespecified protocol registered with the PROSPERO register of systematic reviews (ID CRD42024540553) and followed the Preferred Reporting Items for Systematic Review and Meta‐Analyses (PRISMA) statement (Table S2).

### Search strategy and selection criteria

We conducted a systematic search of the scientific literature in the following databases, covering records from the earliest available record through 31 March 2025: PubMed/MEDLINE, CINAHL and Academic Search Complete via EBSCOhost, Proquest, Web of Science, World Health Organisation (WHO) Global Health Library and Global Index Medicus. The systematic search included key terms ‘diabetes’, ‘glucose intolerance’, ‘dyslipidaemia’, ‘metabolic syndrome’, ‘insulin resistance’, ‘obesity’, ‘hypertension’, ‘cardiovascular disease’ or ‘lipodystrophy’ for health outcomes, paired with ‘Anti‐HIV Agent’, ‘Anti‐HIV drug’, ‘AIDS drug’, ‘nucleoside reverse transcriptase inhibitor’, ‘non‐nucleoside reverse transcriptase inhibitor’, ‘protease inhibitor’ or ‘integrase strand transfer inhibitor’ for exposures. The complete search strategy is found in the Table S1.

Studies were included if they were original quantitative non‐randomized observational research studies published in English that compare ART‐treated and ART‐naïve adult people living with HIV (age ≥ 18 years) and report metabolic outcomes of interest. ART‐naïve adult people living with HIV are people living with HIV who had no history of ART use prior to or who were not yet receiving ART at the time of the outcome assessment. More specifically, we included studies that assessed the prevalence or incidence of metabolic syndrome (using any diagnostic criteria), type 2 diabetes mellitus, impaired fasting glucose, impaired glucose tolerance, dyslipidaemia, hyperlipidaemia, insulin resistance (using any diagnostic criteria), visceral adiposity (using any diagnostic criteria), lipoatrophy and lipodystrophy. We excluded studies focusing on adolescent populations, animal models and articles unrelated to the pharmacological agents or metabolic outcomes of interest.

After deduplication using EndNote, we uploaded search hits to Covidence systematic review software [14]. Two authors (MC and SM) independently performed the literature search, screened the titles and abstracts and assessed the full texts according to the above‐mentioned inclusion and exclusion criteria. Data extraction was performed by two authors (SM and ECG), and two others (MRM and MC) reviewed the extracted data for completeness, performed risk of bias assessment and rated the certainty of evidence. Any arising disagreement was solved by discussion among all authors until consensus was reached.

We deployed the standardized data collection form of the Cochrane Collaboration Public Health Group [15] to extract the following information from each included study: authors, publication date and journal, country, type of study, aims and objectives, sampling techniques and dates of data collection, sample size, age and sex of participants, exposures and outcomes including outcome measures, key conclusions, limitations and recommendations.

Assessment of the risk of bias was subsequently performed by following the Risk Of Bias In Non‐randomized Studies—of Interventions, Version 2 (ROBINS‐I V2) assessment tool [16]. The following seven domains were evaluated and a result matrix was created using the robvis tool [17]: confounding (D1), classification of intervention (D2), selection into the study (D3), deviations from intended intervention (D4), missing data (D5), measurement of the outcome (D6) and selection of reported result (D7). The GRADE approach was further utilized to rate the certainty of evidence in included studies [18].

### Meta‐analysis

Following the Cochrane handbook for systematic reviews [19], we performed a meta‐analysis if there were at least two studies reporting on the same ART combination, provided that those two studies can be meaningfully pooled.

From each eligible study, we extracted the number of participants with and without metabolic complications in both ART‐treated and ART‐naïve groups. Using these data, we calculated study‐specific odds ratios (ORs) with corresponding 95% confidence intervals (CIs), comparing the odds of metabolic outcomes between the two groups. Given that homogeneity of effects across studies is unlikely, the random‐effects model was pre‐specified as the main analytical approach. Fixed‐effect analyses were also performed for comparison. To detect heterogeneity, we used a $\chi^{2}$ test and the $I^{2}$ value, which were considered significant if the $\chi^{2}$ test resulted in <0.1 or if $I^{2}$ was >50% [19]. Prevalence and incidence data were analysed separately, and a separate narrative synthesis will be provided when the number of studies was insufficient for a statistically meaningful pool.

Where at least two studies reported the same ART regimen combination or diagnostic criteria, we conducted subgroup analyses stratified by each. Potential publication bias was evaluated through visual inspection of funnel plots and formally tested using Egger's regression asymmetry test [20]. Finally, sensitivity analyses were performed by sequentially omitting one outlier at a time (leave‐one‐out analysis) to examine the robustness of pooled estimates and by additionally excluding studies with designs other than cross‐sectional. Outliers were defined as any effect size for which the CIs did not overlap with the CI of the pooled effect [21].

Meta‐analyses were conducted in R version 4.4.1 using ‘meta’ package [22]. A two‐sided *p*‐value <0.05 was considered statistically significant.

## RESULTS

**FIGURE 1:**
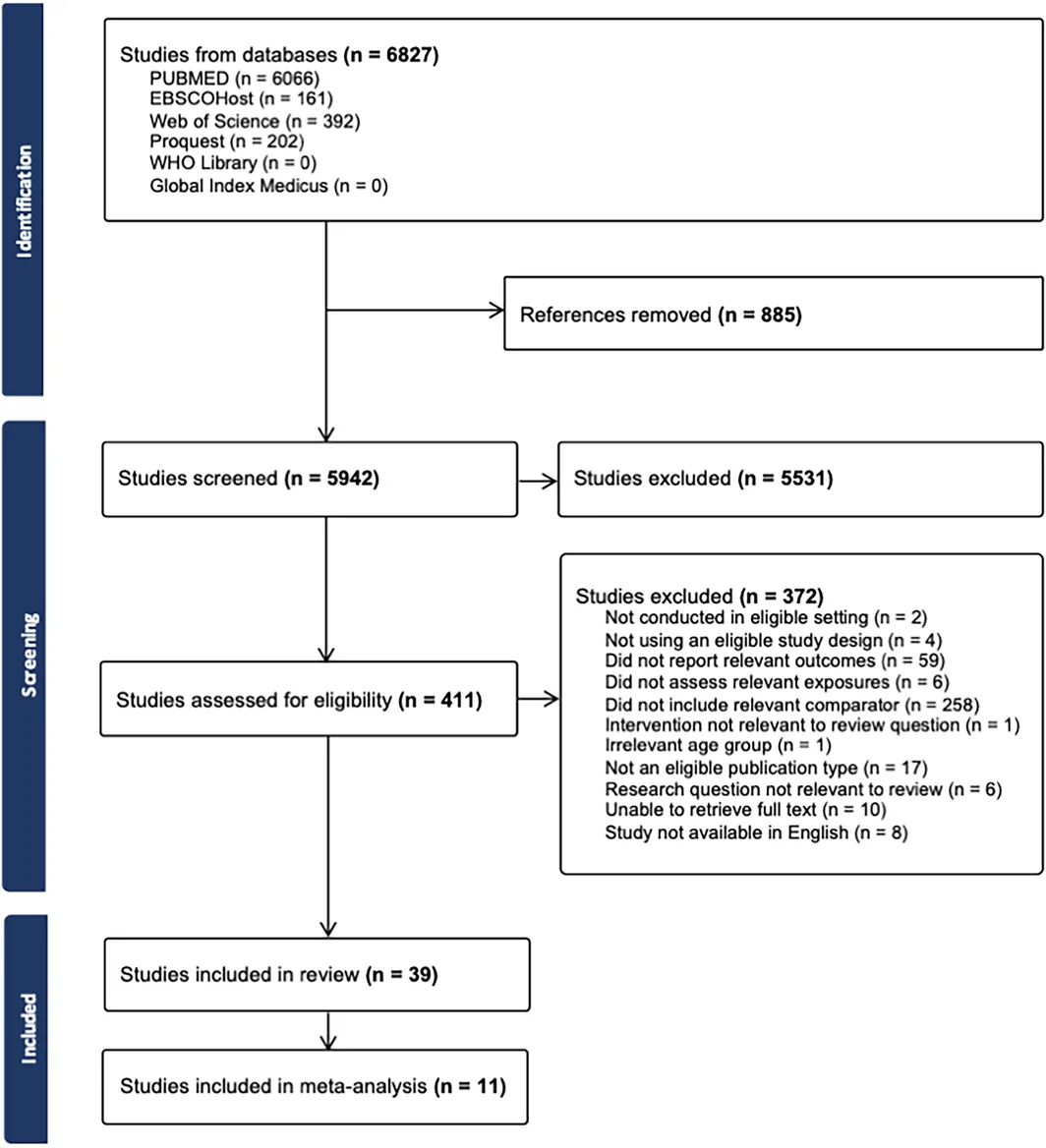
PRISMA flow diagram of study selection. The flowchart depicts the process of literature identification, screening, eligibility assessment and final inclusion in the systematic review and meta‐analysis.

The literature search yielded a total of 5942 non‐duplicate publications for title and abstract screening, in which 5531 were excluded primarily because they did not report metabolic outcomes, were conducted in populations without HIV or were not original research. A total of 411 studies remained for detailed full‐text evaluation (Figure 1). Finally, we included 39 observational non‐randomized studies with information from 24 632 people living with HIV.

**FIGURE 2:**
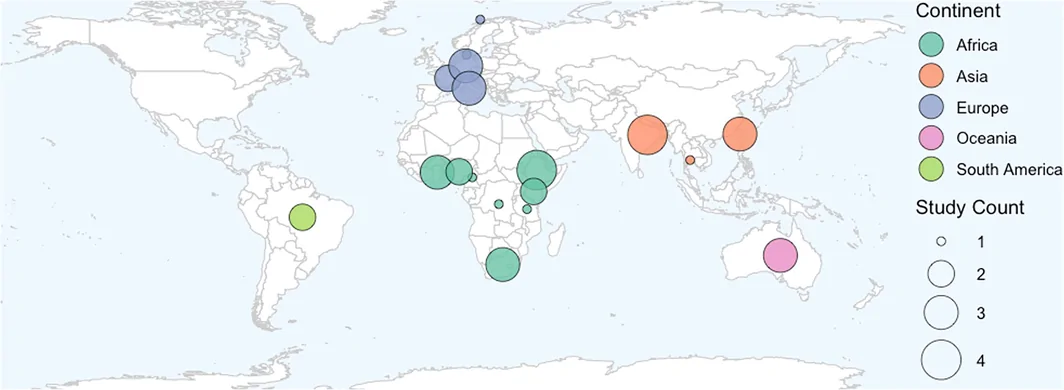
Geographic distribution of studies included in the review. Each circle represents a country with included studies, with circle size proportional to the number of studies conducted. Circle colours indicate the continent: Africa (teal), Asia (orange), Europe (purple), Oceania (pink) and South America (green). The study count legend denotes the number of studies per country (range: 1–4). Countries without eligible studies are not shown.

As illustrated in Figure 2, the 39 included studies were conducted on five different continents: Africa (16), Asia (8), Europe (10), Oceania (3) and South America (2) (Figure 2). In more detail, Table 1 lists the characteristics of these studies which were all published between 1998 and 2025 and included 27 cross‐sectional studies, 2 case–control studies, 9 cohort (3 prospective, 2 retrospective, 4 unspecified) studies and 1 study not reporting the study design. The number of participants per study ranged from 55 to 9594.

**TABLE 1:** Table of characteristics of 39 included studies, sorted by author and country.

No	Study	Study country	Study design	Antiretroviral therapy combination	Active drug regimen (*n*)	Total *n*	Mean age (years)	Follow‐up time	Primary outcome of interest
1	Carr et al. [23]	Australia	Cross‐sectional	Not specified	PI: Indinavir (*n* = 77), Ritonavir + Saquinavir (*n* = 25), Nelfinavir + Saquinavir (*n* = 9), Nelfinavir (*n* = 4), Saquinavir (*n* = 1).	148	PI‐treated (*n* = 116): 40.4 ± 0.8 PI‐naive (*n* = 32): 38.2 ± 1.5	Not applicable	Lipodystrophy, hyperlipidaemia, insulin resistance (estimated using the homeostasis model)
2	Carr et al. [24]	Australia	Case–control	NRTI + PI	NRTI cases (*n* = 14) Abacavir (*n* = 2), Stavudine + Didanosine (*n* = 10), Zidovudine (*n* = 2). Combined NRTI + PI Controls (*n* = 146) (e.g., Abacavir, Stavudine, Didanosine, Zidovudine + Indinavir [*n* = 79], Ritonavir + Saquinavir [*n* = 28], Nelfinavir [*n* = 19], Nelfinavir‐Saquinavir [*n* = 10] and Saquinavir [*n* = 10]). ART‐naïve (*n* = 32). NRTI Controls: (*n* = 28).	220	NRTI cases (*n* = 14): 49.6 ± 1.9 NRTI control (*n* = 28): 41.8 ± 1.6 Combined cases (*n* = 102): 43.4 ± 0.7 Combined control (*n* = 44): 41.1 ± 1.4 ART‐naïve (*n* = 32): 40.4 ± 2.3	2 months	Lipoatrophy
3	Hammond et al. [25]	Australia	Cohort	HAART include NRTI, further details not provided	Thymidine NRTI recipients: (*n* = 15) (Zidovudine, Stavudine). Non‐Thymidine NRTIs: (*n* = 15) (Abacavir, Tenofovir). NRTI‐switching group: (*n* = 28) (Prior Thymidine to Abacavir, Tenofovir).	58	Combined median (*n* = 58): 42 (32–50)	20 months	Lipoatrophy
4	Guimarães et al. [26]	Brazil	Cross‐sectional	Not specified	Specific drugs were not mentioned in the paper. ARV‐treated (*n* = 123) versus ARV‐naïve (*n* = 37)	160	Combined (*n* = 160): 39 ± 9	Not applicable	Visceral adiposity (measured by ultrasound)
5	Soares et al. [27]	Brazil	Cross‐sectional	Not specified	G1:92 HAART with PI (*n* = 70) and without PI (*n* = 22). G2:70 HAART with PI (*n* = 45) and without PI (*n* = 25). G3: No ART (*n* = 65). Specific drugs were not mentioned in the paper.	227	Combined (*n* = 227): range 20–59	Not applicable	Lipodystrophy
6	Ekali et al. [28]	Cameroon	Cross‐sectional	HAART include NRTI, further details not provided	Stavudine (d4T) + Lamivudine (3TC): 27.9% of 44 (1–13 months), 46.9% of 35 (14–33 months), 67.6% of 36 (34–86 months). ART‐naïve (*n* = 28).	143	1–13 months (*n* = 44): 38.0 ± 9.2 14–33 months (*n* = 35): 37.8 ± 9.4 34–86 months (*n* = 36): 45.7 ± 9.7 ART‐naïve (*n* = 28): 35.8 ± 8.3	Not applicable	Insulin resistance (measured by the short insulin tolerance test [SITT])
7	Madone Mandina et al. [29]	Democratic Republic of Congo	Cross‐sectional	2 NRTIs + 1 NNRTI	DRC's Definition of Combination ART (*n* = 49): Stavudine + Lamivudine with Nevirapine or Efavirenz OR Zidovudine + Lamivudine with Nevirapine or Efavirenz (2 NRTIs + 1 NNRTI). ART‐naïve (*n* = 53).	102	With ART (*n* = 49): 43 ± 9.2 ART‐naïve (*n* = 53): 43.6 ± 13.3	Not applicable	Type 2 diabetes mellitus
8	Hansen et al. [30]	Denmark	Cross‐sectional	Not specified	Thymidine NRTIs (stavudine, zidovudine): 55.4% current, 85.2% ever. Non‐Thymidine NRTIs: 82.7% current, 85.9% ever. NNRTIs: 53.5% current, 68.6% ever. PIs: 31.4% current, 63.4% ever.	578	No MS median (*n* = 413): 43.0 (38.4–51.1) With MS median (*n* = 153): 46.7 (40.8–54.6)	Not applicable	Metabolic syndrome (defined by National Cholesterol Education Program Adult Treatment Panel III [NCEP ATP III] criteria)
9	Abebe et al. [31]	Ethiopia	Cross‐sectional	2 NRTIs + 1 NNRTI	On ART = 295: Zidovudine + Lamivudine + Nevirapine = 157 (50.6%). ART‐naïve = 167.	462	With ART (*n* = 259): 37.5 ± 9.2 Pre‐ART (*n* = 167): 34.6 ± 9.9	Not applicable	Type 2 diabetes mellitus
10	Bune et al. [32]	Ethiopia	Cross‐sectional	Not specified	Following the Ethiopian national guideline which includes various first‐line and second‐line (*n* = 422). Specific drugs were not mentioned in the paper. ART‐naïve (*n* = 211).	633	Combined (*n* = 633): 36.4 ± 8.7	Not applicable	Metabolic syndrome (defined by International Diabetes Federation [IDF] criteria)
11	Tadewos et al. [33]	Ethiopia	Cross‐sectional	2 NRTIs + 1 NNRTI	ART‐treated: (*n* = 113). 22 (19.5%) patients on ZDV/3TC/EFV; 36 (31.8%) patients on ZDV/3TC/NVP; 24 (21.2%) patients on d4T/3TC/EFV; 31 (27.4%) patients on d4T/3TC/NVP. ART‐naïve (*n* = 113).	226	ART‐treated (*n* = 113): 37.2 ± 8.7 ART‐naïve (*n* = 113): 33.7 ± 8.3	Not applicable	Dyslipidaemia
12	Tesfaye et al. [34]	Ethiopia	Cross‐sectional	2 NRTIs + 1 NNRTI	ART‐treated: (*n* = 188). 38 patients on ZDV/3TC/NVP (38.8% of 73 ZDV‐based); 35 patients on ZDV/3TC/EFV (remaining of 73 ZDV‐based); 47 patients on TDF/3TC/EFV (34% of 62 TDF‐based); 15 patients on TDF/3TC/NVP (remaining of 62 TDF‐based); 27 patients on d4T/3TC/EFV (14.4%); 26 patients on d4T/3TC/NVP (13.8%). ART‐naïve (*n* = 186).	374	ART‐treated (*n* = 188): 32.7 ± 9.7 ART‐naïve (*n* = 186): 32.6 ± 7.8	Not applicable	Metabolic syndrome (defined by NCEP ATP III criteria and the IDF criteria)
13	Capeau et al. [35]	France	Prospective cohort	Not specified	NRTI (prior to PI): Stavudine (79% of patients exposed), Zidovudine (90% of patients exposed), Didanosine (68% of patients exposed), PI: Indinavir (54% of patients exposed), Nelfinavir (56% of patients exposed).	1046	Median: Normal (*n* = 374): 34.4 (30.0–39.4) IGT (*n* = 450): 36.7 (33–43.3) Once (*n* = 111): 38.1 (33.2–45.3) Diabetic (*n* = 111) 43 (38.1–51.7)	12 and 20 months	Type 2 diabetes mellitus
14	Saint‐Marc et al. [36]	France	Cross‐sectional	HAART include NRTI, further details not provided	ART‐treated: (*n* = 43) Stavudine + lamivudine (*n* = 14); Stavudine + didanosine (*n* = 13); Zidovudine + didanosine (*n* = 13); Zidovudine + lamivudine (*n* = 2); Zidovudine + zalcitabine (*n* = 1). ART‐naïve (*n* = 15).	58	ART‐treated (*n* = 43): 41.1 ± 10.9 (pooled) ART‐naïve (*n* = 15): 39.7 ± 6.8	Not applicable	Lipodystrophy
15	Blass et al. [37]	Germany	Cross‐sectional	2 NRTIs + 1 NNRTI	ART‐treated (*n* = 28). PI‐based: (*n* = 19) Lopinavir/Ritonavir (*n* = 16); Atazanavir (*n* = 3). NRTI + NNRTI: (*n* = 9) AZT + 3TC + NNRTI combinations; TDF‐based combinations; Various NRTI combinations including 3TC, ABC, FTC, d4T with EFV or NVP. ART‐naïve (*n* = 16). Healthy control (*n* = 11).	55	Median PI‐ART (*n* = 19): 44 (29–68) Non‐PI‐ART (*n* = 9): 39 (34–59) ART‐naïve (*n* = 16): 37 (24–45) Healthy (*n* = 11): 33 (24–58)	Not applicable	Impaired fasting glucose, impaired glucose tolerance, insulin resistance, basal c‐peptide, hyperinsulinaemia, new onset diabetes mellitus
16	Jin et al. [38]	Germany	Retrospective cohort	2 NRTIs + 1 PI 2 NRTIs + 1 NNRTI 2 NRTIs + 1 INSTI	ART‐treated: (*n* = 153) NRTI: AZT/3TC combinations; Tenofovir; Lamivudine; d4T; FTC/TDF; Abacavir; 2NRTIs + PI (*n* = 37): Lopinavir/Ritonavir; 2NRTIs + NNRTI (*n* = 68): Efavirenz, Nevirapine; 2NRTIs + Integrase Inhibitor (*n* = 28); Other regimens (*n* = 20). ART‐naïve (*n* = 27).	180	ART‐treated (*n* = 153): 48.2 ± 10.8 ART‐naïve (*n* = 27): 44.7 ± 11.9	Not reported	Type 2 diabetes mellitus
17	Schmidt et al. [39]	Germany	Prospective cohort	2 NRTIs + 1 PI	PI‐based: Ritonavir, Saquinavir, Indinavir; Nelfinavir (*n* = 98). PI‐naïve (*n* = 23). ART‐naïve (*n* = 10).	131	T IV/V (*n* = 32): 44.2 ± 10.6 T IIa (*n* = 6): 54.2 ± 6.6 T IIb (*n* = 18): 53.4 ± 11.4 Normal (*n* = 42): 45.9 ± 12.7	Not reported	Hyperlipidaemia
18	Dzudzor et al. [40]	Ghana	Case–control	2 NRTIs + 1 NNRTI	ART‐treated: (*n* = 158). LPV/r‐based (*n* = 12, 7.6%); TDF/3TC/NVP or EFV (*n* = 94, 59.5%); AZT/3TC/NVP or EFV (*n* = 52, 32.9%). ART‐naïve (*n* = 150). Non‐HIV control (*n* = 156).	464	ART‐treated (*n* = 158): 39 ± 11.4 ART‐naïve (*n* = 150): 38.2 ± 11.6 Non‐HIV (*n* = 156): 36.7 ± 14.4	Not reported	Metabolic syndrome (defined by the criteria of the Joint Interim Statement [JIS], NCEP ATP III and IDF)
19	Ngala and Fianko [41]	Ghana	Cross‐sectional	2 NRTIs + 1 NNRTI	ART‐treated: (*n* = 164). d4T/3TC/NVP (*n* = 68); d4T/3TC/EFV (*n* = 30); AZT/3TC/NVP (*n* = 36); AZT/3TC/EFV (*n* = 21). ART‐naïve (*n* = 141).	305	ART‐treated (*n* = 164): 38.1 ± 0.65 ART‐naïve (*n* = 141): 38.8 ± 0.76	Not applicable	Dyslipidaemia, impaired fasting glucose, type 2 diabetes mellitus, lipoatrophy, lipohypertrophy
20	Lartey et al. [42]	Ghana	Cohort	2 NRTIs + 1 INSTI	Dolutegravir‐based regimen (Dolutegravir is combined with either TDF/3TC or ABC/3TC).	1334	Distribution <25 years: 53 (3.97%) 25–49 years: 852 (63.87%) 50–59 years: 277 (20.76%) >60 years: 152 (11.39%)	12, 24, 36 and 72 weeks	Type 2 diabetes mellitus
21	Idiculla et al. [43]	India	Cross‐sectional	2 NRTIs 2 NRTIs + 1 NNRTI	ART‐treated (*n* = 76). ZDV/3TC‐based (*n* = 48); d4T/3TC‐based (*n* = 26); TDF/3TC‐based (*n* = 2) with EFV or NVP. ART‐naïve (*n* = 71). Non‐HIV control (*n* = 70).	217	ART‐treated (*n* = 76): 35.9 ± 6.9 ART‐naïve (*n* = 71): 34.2 ± 7.6 Non‐HIV (*n* = 70): 32.2 ± 7.5	Not applicable	Metabolic syndrome (defined by NCEP ATP III criteria)
22	Indumati et al. [44]	India	Cross‐sectional	2 NRTIs + 1 NNRTI	ART‐treated (*n* = 100). SLN regimen (d4T/3TC/NVP, *n* = 59); ZLN regimen (AZT/3TC/NVP, *n* = 41). ART‐naïve (*n* = 100).	200	ART‐treated (*n* = 100): 35.7 ± 9.0 ART‐naïve (*n* = 100): 36.0 ± 8.9	Not applicable	Dyslipidaemia
23	Kalyanasundaram et al. [45]	India	Cross‐sectional	2 NRTIs + 1 NNRTI	ART‐treated (*n* = 145). AZT/3TC/NVP (*n* = 54, 37.8%); AZT/3TC/EFV (*n* = 4, 2.8%); d4T/3TC/NVP (*n* = 83, 58%); d4T/3TC/EFV (*n* = 2, 1.4%); Unspecified regimen (*n* = 2). ART‐naïve (*n* = 146). Non‐HIV control (*n* = 72).	363	ART‐treated (*n* = 145): 35.6 ± 7.9 ART‐naïve (*n* = 146): 31.9 ± 6.4 Non‐HIV (*n* = 72): 34.4 ± 6.3	Not applicable	Dyslipidaemia
24	Sreekantamurthy et al. [46]	India	Cross‐sectional	2 NRTIs + 1 NNRTI	ART‐treated (*n* = 79). AZT‐based (AZT/3TC/NVP or EFV, *n* = 22); d4T‐based (d4T/3TC/NVP or EFV, *n* = 18); TDF‐based (TDF/3TC/NVP or EFV, *n* = 15); PI‐based (*n* = 24) *Atazanavir/Ritonavir. ART‐naïve (*n* = 22).	101	Range ART‐treated (*n* = 79): 26–60 ART‐naïve (*n* = 22): 24–51	Not applicable	Lipoatrophy
25	Bonfanti et al. [47]	Italy	Prospective cohort	Not specified	Comparing NNRTI‐based and PI‐based regimens, but specific drugs were not mentioned in the paper.	188	Median with MS (*n* = 14): 47 (33–53) Without MS (*n* = 174): 38 (32–45)	3 years	Metabolic syndrome (defined by NCEP ATP III criteria)
26	Calza et al. [48]	Italy	Cross‐sectional	2 NRTIs + 1 NNRTI 2 NRTIs + 1 PI 2 NRTIs + 1 INSTI	ART‐treated (*n* = 488). 2 NRTIs + 1 NNRTI (Rilpivirine, Efavirenz) = 219; 2 NRTIs + 1 PI (Darunavir/Ritonavir; Atazanavir/Ritonavir) = 167; 2 NRTIs + 1 INSTI (Raltegravir, Elvitegravir/Cobicistat, Dolutegravir) = 102. ART‐naïve (*n* = 98).	586	ART‐treated (*n* = 488): 46.7 ± 18.5 ART‐naïve (*n* = 98): 44.1 ± 15.9	Not applicable	Metabolic syndrome (defined by NCEP ATP III criteria and IDF criteria)
27	Manfredi and Chiodo [49]	Italy	Retrospective cohort	2 NRTIs + 1 PI 2 NRTIs + 1 NNRTI	ART‐treated (*n* = 160). 2 NRTIs + Saquinavir (hard gel formulation) = 40; 2 NRTIs + Indinavir = 40; 2 NRTIs + Ritonavir = 40; 2 NRTIs with NNRTI (13 patients) or without NNRTI (27 patients). ART‐naïve (*n* = 40).	200	No data on age, its mean, median, range or distribution.	3 months	Dyslipidaemia
28	Manuthu et al. [50]	Kenya	Cross‐sectional	2 NRTIs + 1 NNRTI	ART‐treated (*n* = 134). d4T + 3TC + NVP (stavudine + lamivudine + nevirapine) − 51.1% of patients; d4T + 3TC + EFV (stavudine + lamivudine + efavirenz) − 31.6% of patients; AZT + 3TC + NVP (zidovudine + lamivudine + nevirapine) − 3.8% of patients; AZT + 3TC + EFV (zidovudine + lamivudine + efavirenz) − 13.5% of patients. ART‐naïve (*n* = 161).	295	ART‐treated (*n* = 134): mean = 39.4, median = 36.5 ART‐naïve (*n* = 161): mea*n* = 36.5, median = 36	Not applicable	Dyslipidaemia, impaired fasting glucose, impaired glucose tolerance, type 2 diabetes mellitus
29	Osoti et al. [51]	Kenya	Cross‐sectional	2 NRTIs + 1 NNRTI	ART‐treated (*n* = 164). 2 NRTI + 1 NNRTI‐based: Tenofovir/lamivudine/nevirapine (56%); Tenofovir/lamivudine/efavirenz (36%). PI‐based: lopinavir/ritonavir (6.7%). ART‐naïve (*n* = 136).	300	ART‐treated (*n* = 164): 43.1 ± 9.4 ART‐naïve (*n* = 136): 37.9 ± 8.9	Not applicable	Metabolic syndrome (defined by the IDF criteria)
30	Muhammad et al. [52]	Nigeria	Cross‐sectional	2 NRTIs + 1 NNRTI	ART‐treated (*n* = 150). 2 NRTI + 1 NNRTI‐based: AZT + 3TC + NVP: 43.3%; AZT + 3TC + EFV: 10%; TDF + 3TC + NVP: 10.7%; TDF + 3TC + EFV: 14%; TDF + FTC + NVP: 10%; TDF + FTC + EFV: 2%. PI‐based 2nd line (LPV/r + NRTI): 4.7%; PI‐based 2nd line (LPV/r + Raltegravir): 5.3%. ART‐naïve (*n* = 150).	300	ART‐treated (*n* = 150): 35.7 ± 10.0 With MS (*n* = 29): 42.3 ± 8.5 Without MS (*n* = 121): 34.1 ± 9.7 ART‐naïve (*n* = 150): 34.0 ± 9.7	Not applicable	Metabolic syndrome (defined by the NCEP ATP III criteria)
31	Ogunmola et al. [53]	Nigeria	Cross‐sectional	2 NRTIs + 1 NNRTI 2 NRTIs + 1 PI	ART‐treated (*n* = 130). Zidovudine, lamivudine, nevirapine (98% of patients), Tenofovir, lamivudine, boosted lopinavir (2% of patients). ART‐naïve (*n* = 120). Non‐HIV control (*n* = 153).	403	ART‐treated (*n* = 130): 38.6 ± 8.0 ART‐naïve (*n* = 120): 36.5 ± 9.1 Non‐HIV (*n* = 153): 35.5 ± 7.6	Not applicable	Obesity (defined by the standardized definition of the World Health Organization: body mass index > = 30 kg/m2)
32	Bergersen et al. [54]	Norway	Cohort	Not specified	ART‐treated (*n* = 207). NRTIs: Stavudine (47%), Lamivudine (85%). NNRTIs: Nevirapine (34%), Efavirenz (4%). PIs: Indinavir (25%), Nelfinavir (25%). ART‐naïve (*n* = 56). Non‐HIV control (*n* = 94).	357	ART‐treated (*n* = 207): 43.4 ± 9.4 ART‐naïve (*n* = 120): 42.8 ± 11.0 Non‐HIV (*n* = 94): 48.8 ± 5.4	Not reported	Metabolic syndrome (criteria used not reported), lipodystrophy, insulin resistance (estimated using the homeostasis model)
33	Dave et al. [55]	South Africa	Cross‐sectional	2 NRTIs + 1 NNRTI	ART‐treated: (*n* = 443). 2 NRTIs + EFV (*n* = 238); 2 NRTIs + NVP (*n* = 205). NRTI backbone: d4T/3TC (66.8%), AZT/3TC (33.2%). ART‐naïve: (*n* = 406).	849	Median with dysglycaemia (*n* = 203): 37 (32–44) Normoglycaemia (*n* = 646): 32 (28–38)	Not applicable	Prediabetes (impaired glucose tolerance or impaired fasting glucose), type 2 diabetes mellitus
34	Levitt et al. [56]	South Africa	Cross‐sectional	2 NRTIs + 1 NNRTI 2 NRTIs + 1 PI	First‐line ART (*n* = 439): d4T/3TC (*n* = 294) or AZT/3TC (*n* = 145) + EFV or NVP. Second‐line ART (*n* = 108): AZT/ddI/LPV‐r (majority of *n* = 108). ART‐naïve (*n* = 393). Community comparison (*n* = 880).	1820	ART‐1st line (*n* = 439): 36.1 ± 8.8 ART‐2nd line (*n* = 108): 36.6 ± 8.3 ART‐naïve (*n* = 393): 33.6 ± 8.7 Non‐HIV (*n* = 880): 39.9 ± 10.0	Not applicable	Impaired glucose tolerance, impaired fasting glucose, type 2 diabetes mellitus
35	Lin et al. [57]	Taiwan	Cohort	2 NRTIs + 1 NNRTI 2 NRTIs + 1 PI	HAART cohort (*n* = 4797) a/t Taiwan's Guideline. 2 NRTIs + NNRTI, 2 NRTIs + PI (boosted & upboosted). Specific drugs were not mentioned in the paper. Non‐HAART cohort (*n* = 4797).	9594	ART‐treated (*n* = 4797): 32.9 ± 8.5 ART‐naïve (*n* = 4797): 32.9 ± 8.5	10 years	Type 2 diabetes mellitus
36	Lu et al. [58]	Taiwan	Cross‐sectional	2 NRTIs + 1 NNRTI 2 NRTIs + 1 PI 2 NRTIs + 1 INSTI	ART‐treated (*n* = 155). NRTIs: AZT, 3TC, ABC, TDF/FTC; combined with NNRTIs: NVP, EFV, RPV, EFV/TDF/FTC (*n* = 72); or PIs: ATZ, LPV/r, DRV (*n* = 62); or InSTIs: RAL (*n* = 19). ART‐naïve (*n* = 45).	200	ART‐treated (*n* = 155): 33.6 ± 8.2 ART‐naïve (*n* = 45): 30.5 ± 7.6	Not applicable	Metabolic syndrome (criteria used not reported)
37	Tu et al. [59]	Taiwan	Cross‐sectional	STR‐Kombi + 2 NRTIs 2 NRTIs + INSTI 2 NRTIs + 1 NNRTI 2 NRTIs + 1 PI	BIC/FTC/TAF (*n* = 300); ABC/3TC/DTG (*n* = 186); EVG/COBI/FTC/TAF (*n* = 28); DTG/RPV, 3TC/DTG (*n* = 144). TDF/FTC/EFV, TDF/3TC/DOR, TAF/FTC/RPV (*n* = 144). ZDV/3TC/DRV/c (*n* = 7).	809	With MetS (*n* = 81): 43.4 ± 10.7 No MetS (*n* = 728): 39.0 ± 10.3	Not applicable	Metabolic syndrome (defined by NCEP ATP III criteria)
38	Maganga et al. [60]	Tanzania	Cross‐sectional	Not specified	ART‐treated (*n* = 150). 18 (12.0%) taking PI (lopinavir/ritonavir). Zidovudine, stavudine, tenofovir, efavirenz and nevirapine uses were categorized and mentioned in regression results. ART‐naïve (*n* = 151). Non‐HIV control (*n* = 153).	454	Median ART‐treated (*n* = 150): 40 (38–47) ART‐naïve (*n* = 152): 37 (32–44) Non‐HIV (*n* = 153): 38 (32–46)	Not applicable	Impaired glucose tolerance, impaired fasting glucose, type 2 diabetes mellitus
39	Jantarapakde et al. [61]	Thailand	Cross‐sectional	Not specified	ART‐treated (*n* = 410). NRTI backbone: lamivudine, stavudine, zidovudine, tenofovir, didanosine and abacavir. NNRTI‐based regimens: (nevirapine and efavirenz): 323 patients (78.8%). PI‐based regimens: indinavir/ritonavir, lopinavir/ritonavir, atazanavir/ritonavir: 61 patients (14.9%). Missing ART regimen data: 26 patients (6.3%). ART‐naïve (*n* = 170).	580	Median ART‐treated (*n* = 410): 39 (34–44) ART‐naïve (*n* = 170): 34 (29–40)	Not applicable	Metabolic syndrome (defined by the criteria of the National Heart Lung and Blood Institute and American Heart Association [NHLBI and AHA])

Abbreviations: ART, antiretroviral therapy; ARV, Antiretroviral therapy; IGT, impaired glucose tolerance; INSTI, integrase strand transfer inhibitor; MS, metabolic syndrome; NNRTI, non‐nucleoside reverse transcriptase inhibitor; NRTI, nucleoside reverse transcriptase inhibitor; PI, protease inhibitor.

Most reported ART combinations included 2 NRTIs + 1 NNRTI (21 studies), followed by 2 NRTIs + 1 PI (10 studies) and 2 NRTIs + 1 INSTI (six studies). A total of 13 studies did not specify exact combinations but referenced general HAART or ART use. Several studies included overlapping ART classes; therefore, the counts reflect reported presence, not exclusive use.

**FIGURE 3:**
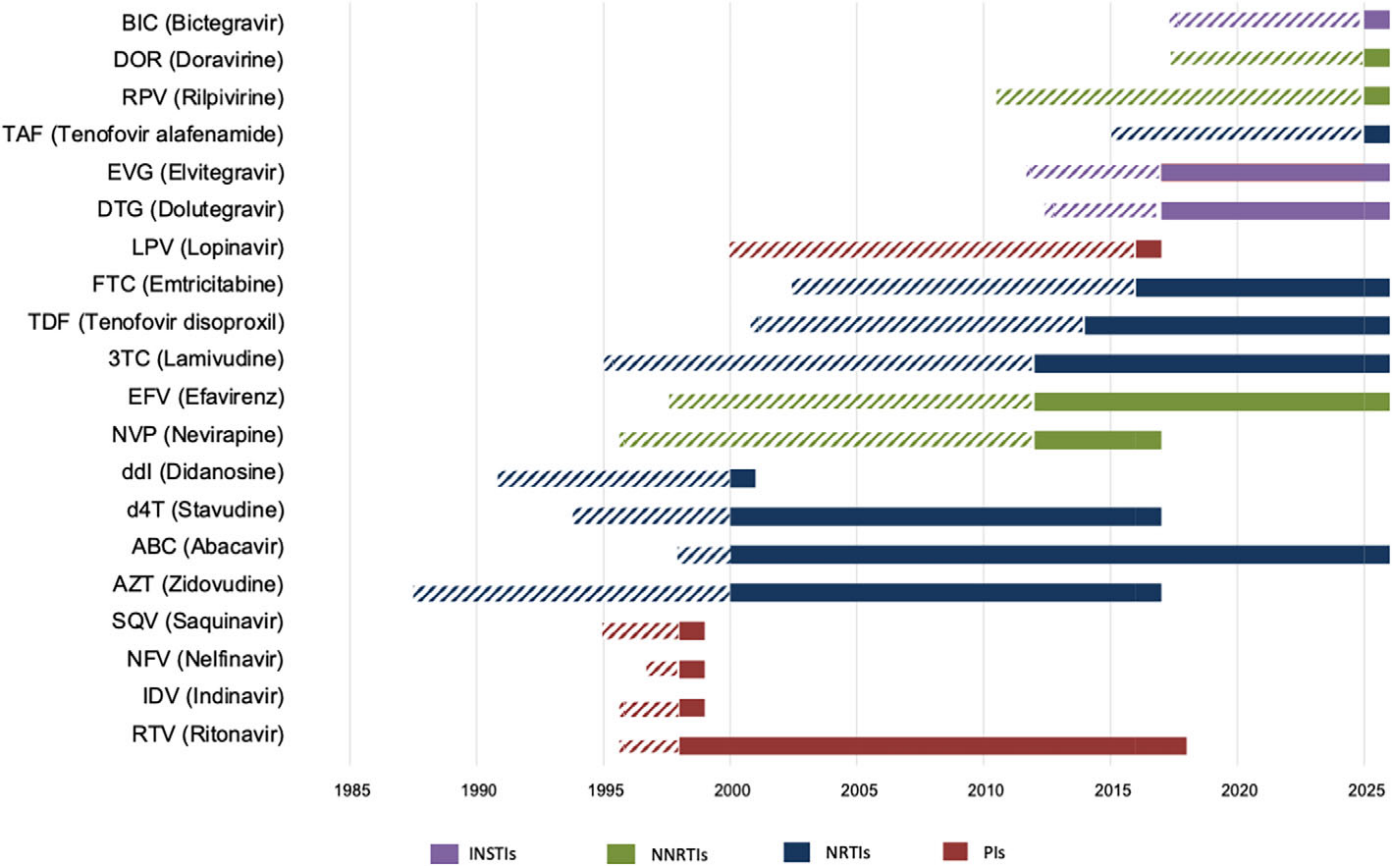
Timeline of antiretroviral therapy (ART) rollout and included studies‐reported drug regimens by ART class. Columns with diagonal‐hatched shading denote the FDA‐approved year of ART rollout. Colour‐filled columns denote the year in which the included studies first reported use of that specific ART. INSTIs, integrase strand transfer inhibitors; NNRTIs, non‐nucleoside reverse transcriptase inhibitors; NRTIs, nucleoside reverse transcriptase inhibitors; PIs, protease inhibitors.

Reported use of ART classes was examined by year of publication, as illustrated in Figure 3. NRTIs formed the backbone of every regimen and appeared throughout the review period, reflecting WHO recommendations that a dual‐NRTI backbone should be combined with a third active agent in most first‐line regimens [62]. NNRTIs were prominent in studies published from the early 2000s until around 2014. PIs dominated the earliest literature (1998–2014) but appeared only sporadically thereafter. The first study in this review containing an INSTI was published in 2016, and the frequency of INSTI‐based regimens rose steadily through 2025.

Over time, the focus of research on metabolic outcomes of ART has shifted considerably. In the early period (1998–2004), study outcomes were dominated by lipid abnormalities, including lipodystrophy, hyperlipidaemia and dyslipidaemia. Between 2005 and 2014, the scope expanded to broader cardiometabolic outcomes, with increasing attention to metabolic syndrome and insulin resistance, while lipid abnormalities continued to feature prominently. In the most recent decade (2015–2025), the literature has centred on weight‐related complications, particularly obesity, excess weight gain, impaired fasting glucose, impaired glucose tolerance, type 2 diabetes mellitus and metabolic syndrome. This transition parallels the global adoption of INSTIs and the growing body of evidence linking these agents to weight gain.

**FIGURE 4:**
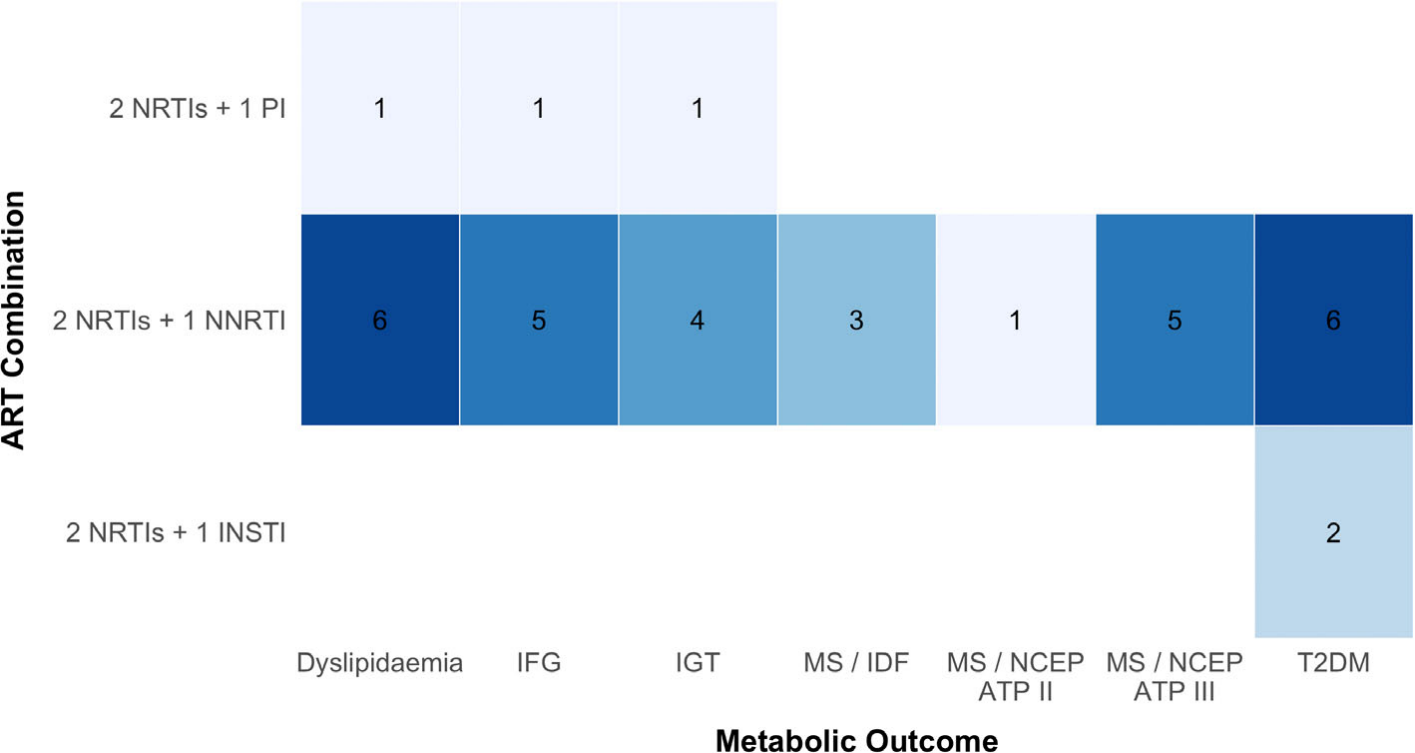
Heatmap of reported metabolic outcomes associated with antiretroviral therapy (ART) combinations. The figure summarizes the number of studies linking specific ART regimens with selected metabolic outcomes. Darker shading corresponds to a higher number of studies reporting the outcome, whereas lighter shading reflects fewer reports. IFG, impaired fasting glucose; IGT, impaired glucose tolerance; INSTI, integrase strand transfer inhibitor; MS‐IDF, metabolic syndrome (International Diabetes Federation criteria); MS‐JIS/NCEP ATP II, metabolic syndrome (Joint Interim Statement/National Cholesterol Education Program Adult Treatment Panel II criteria); MS‐NCEP ATP III, metabolic syndrome (National Cholesterol Education Program Adult Treatment Panel III criteria); NNRTI, non‐nucleoside reverse transcriptase inhibitor; NRTI, nucleoside/nucleotide reverse transcriptase inhibitor; PI, protease inhibitor; T2DM, type 2 diabetes mellitus.

Further, metabolic syndrome and type 2 diabetes mellitus were included as outcomes in the meta‐analysis as they were reported by at least two studies, provided that those two studies can be meaningfully pooled (Figure 4).

**FIGURE 5:**
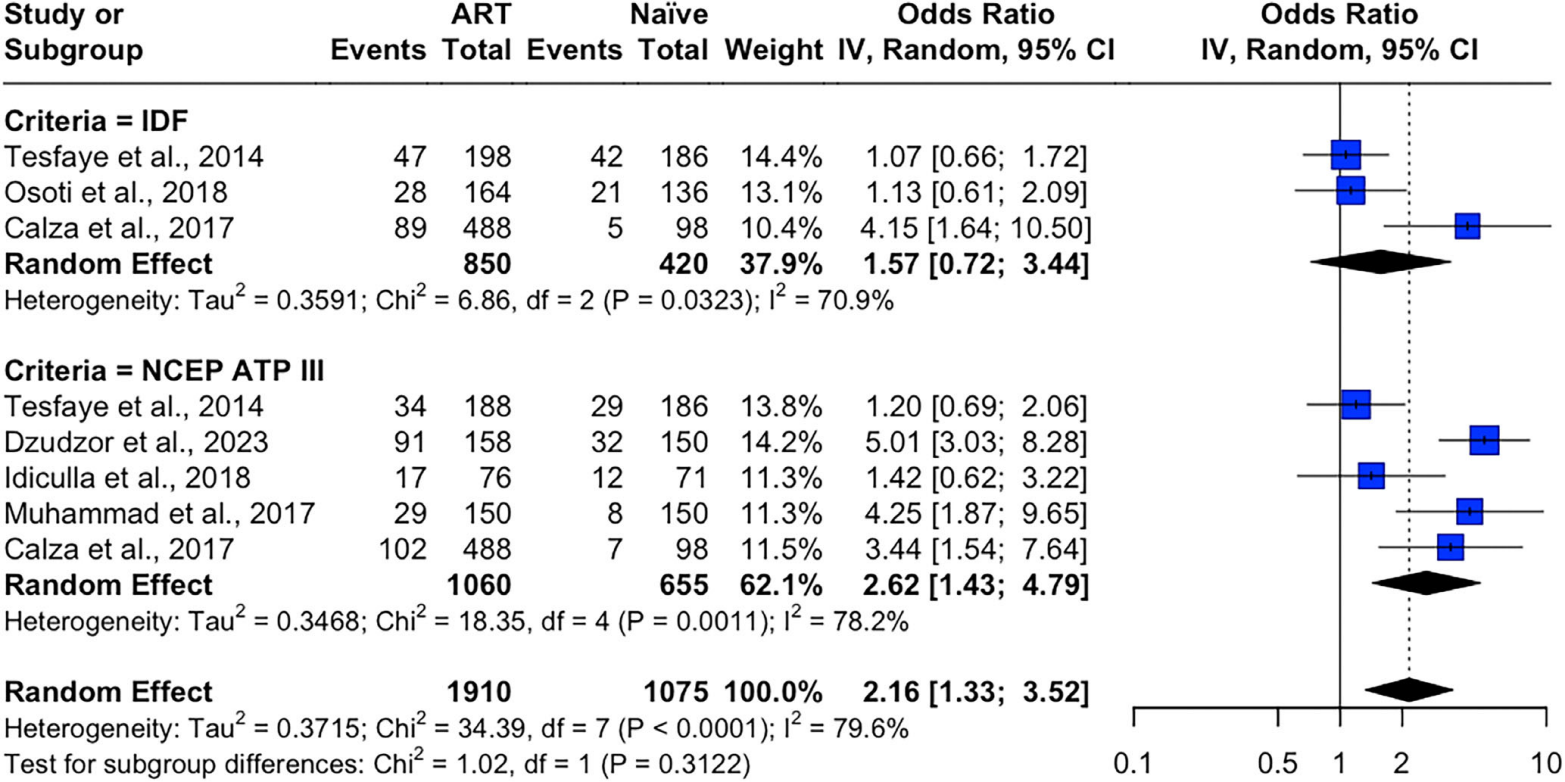
Association of antiretroviral therapy (ART)‐treated versus ART‐Naïve people living with HIV with prevalence of metabolic syndrome. CI, confidence interval; IDF, International Diabetes Federation criteria; NCEP ATP III, National Cholesterol Education Program Adult Treatment Panel III criteria.

Data on prevalence of metabolic syndrome were available from a total of six studies including 1910 ART‐treated people living with HIV (437 cases) and 1075 ART‐naïve people living with HIV (156 cases) with summary for combined criteria OR = 2.16 (95% CI = 1.33–3.52), *I* ^2^ = 79.6% (Figure 5). These six studies evaluated ART regimens consisting of 2 NRTIs combined with 1 NNRTI. Consequently, the analysis was not stratified by regimen type.

**FIGURE 6:**
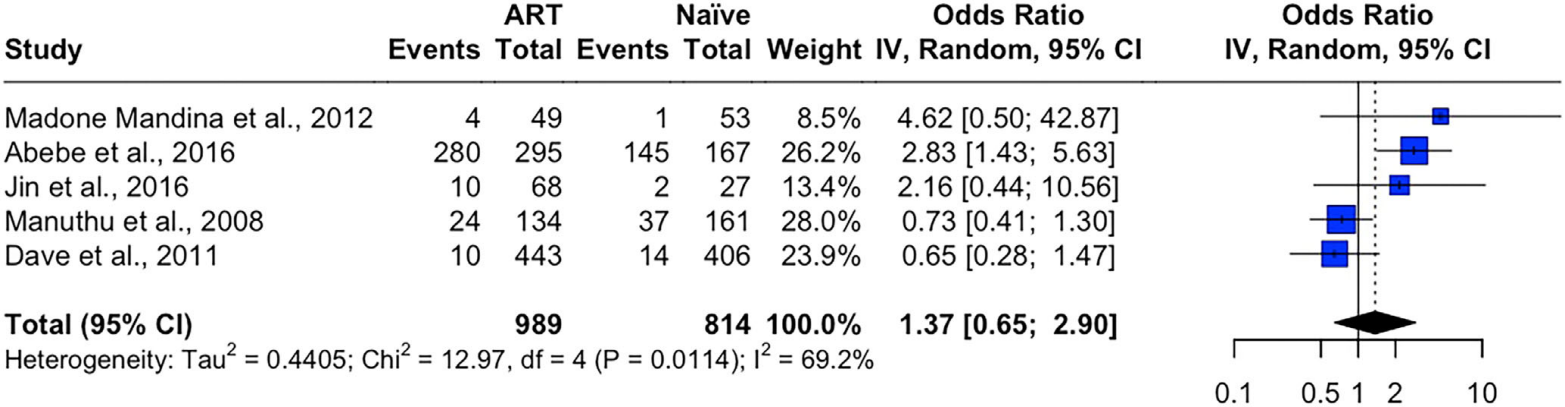
Association of antiretroviral therapy (ART)‐treated versus ART‐Naïve people living with HIV with prevalence of type 2 diabetes mellitus in those treated with ART combination 2 NRTIs + 1 NNRTI. CI, confidence interval.

For type 2 diabetes mellitus, a total of five studies reported the use of 2 NRTIs + 1 NNRTI combination were included (Figure 6). Data on prevalence of type 2 diabetes were available for 989 ART‐treated people living with HIV (328 cases) and 814 ART‐Naïve people living with HIV (199 cases) with summary OR = 1.37 (95% CI = 0.65–2.90), *I* ^2^ = 69.2%. The pooled analysis indicated no significant difference in the odds of having type 2 diabetes mellitus among individuals receiving ART compared to ART‐naïve individuals.

The risk of bias assessment across various categories yielded heterogeneous outcomes revealing the highest concerns in the domains of confounding (D1) and participant selection (D2), primarily due to inadequate control for confounders and unclear inclusion criteria (see Figures S3 and S4). Statistical analysis and reporting (D6) also showed moderate concerns in a subset of studies related to incomplete reporting or unclear methods.

In contrast, most studies demonstrated low risk in the domains of intervention classification (D3), deviations from intended interventions (D4) and missing data (D5), though some moderate risks were noted. Overall, while most studies were rated with low risk across domains, concerns about residual confounding and the lack of confounder adjustment in some studies raise concerns about confounding bias and warrant cautious interpretation of their findings.

Using GRADE, we rated the overall evidence for both meta‐analyses as low because of study bias, inconsistent effect sizes, small subgroup samples and likely publication bias (see Table S3).

**FIGURE 7:**
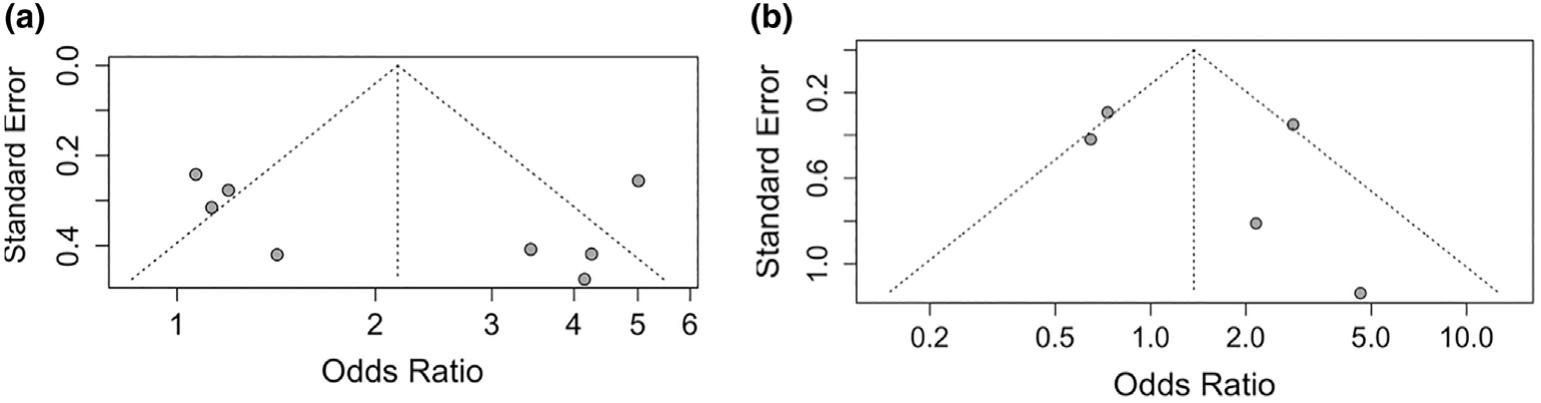
Funnel plots for publication bias. The odds ratio (OR) is on the *x*‐axis and the standard error of the log(OR) on the *y*‐axis; the vertical line marks the pooled effect. (a) Metabolic syndrome; (b) Type 2 diabetes mellitus.

For both outcomes, funnel plots showed right‐sided asymmetry (Figure 7). With less than 10 studies per outcome, Egger's tests are underpowered, and between‐study heterogeneity could also generate asymmetry. Overall, evidence for publication bias remains inconclusive.

Two studies reported incident diabetes relevant to ART exposure. Lin et al. [57] directly compared ART‐treated (mainly consisting of 2 NRTIs plus NNRTI or PIs) and ART‐naïve people living with HIV and observed a higher diabetes incidence among those receiving ART (10‐year cumulative incidence 7.16% vs. 2.24%), with ART independently associated with increased risk (adjusted HR 2.39, 1.65–3.45). Lartey et al. [42] followed ART‐naïve and ART‐experienced people living with HIV who all initiated dolutegravir‐based ART and reported substantial diabetes incidence after ART initiation. However, the absence of a group remaining ART‐naïve during follow‐up precluded direct comparison with ART non‐use.

In sensitivity analysis, we excluded studies with designs other than cross‐sectional study design for both outcomes. The pooled estimates remained consistent, indicating that the overall findings were robust to the exclusion of these studies (see Figures S1 and S2).

With regard to differences between the protocol and the systematic review, some methods in the protocol could not be applied due to insufficient data or a lack of information from the articles included in the review. In the original protocol, we also anticipated performing meta‐analysis for each of the prespecified outcomes for both cardiometabolic outcomes and cardiometabolic clinical measures. However, due to constraints related to the available time frame and resource capacity, we could only undertake systematic review and meta‐analysis for the metabolic outcomes.

## DISCUSSION

In this study, we examined the association between specific ART regimens and metabolic outcomes, and we observed that the safety spectrum has evolved over time in parallel with the predominant use of certain drugs and combinations. The combinations of NRTI and PI were linked to lipodystrophy and dyslipidaemia, whereas INSTI‐based and selected dual‐drug strategies are more frequently associated with metabolic outcomes such as obesity, excess weight gain, type 2 diabetes mellitus and metabolic syndrome. The meta‐analysis showed a significant concern: ART‐treated adult people living with HIV have roughly double the odds of developing metabolic syndrome compared to ART‐naïve adult people living with HIV. In contrast, our meta‐analytic evidence did not demonstrate a statistically significant association between ART use and the odds of developing type 2 diabetes mellitus.

Changes in the age distribution of people living with HIV are central to interpreting metabolic risk over time. Across included studies, participant age varied by calendar year, with more recent studies enrolling younger populations, while ART‐treated groups tended to be older than those not yet receiving ART. Given the strong association between age and cardiometabolic outcomes, this heterogeneity may contribute to observed differences in metabolic risk independent of ART effects. Although many studies adjusted for age, residual confounding cannot be excluded. Accordingly, pooled estimates likely reflect the combined influence of age structure, ART exposure and temporal differences in treatment practices and study design rather than isolated effects of ART alone.

Compared with a recent global meta‐analysis which reported a 60% increase in metabolic syndrome risk in HIV‐infected individuals compared to non‐HIV‐infected individuals (pooled OR = 1.60, 95% CI = 1.15–2.23), and a 50% increase in metabolic syndrome risk in ART‐treated compared to ART‐naïve people living with HIV (pooled OR = 1.50, 95% CI = 1.22–1.86), our pooled estimate was higher [63].

Our stratified analysis of type 2 diabetes mellitus was restricted to NRTI/NNRTI combinations, as the number of eligible studies reporting other combinations was insufficient to support statistically meaningful pooling. Two observational studies suggest a potential risk of type 2 diabetes mellitus following INSTI initiation [38, 42], particularly with dolutegravir‐based therapy [42]. These findings align with studies that implicate INSTI regimens having the stronger metabolic influence: the RESPOND consortium of cohorts in Europe and Australia reported a 48% higher incidence rate of type 2 diabetes mellitus with current INSTI use compared to non‐INSTIs [64], and NA‐ACCORD in North America found a similar excess risk when first‐line INSTIs were compared with NNRTI backbones after adjustment for weight gain [65].

Our study shows that the increased metabolic risks associated with ART warrant a targeted follow‐up for people living with HIV. The twofold risk estimates denote the need for routine cardiometabolic monitoring as integral to HIV care, particularly in low‐ and middle‐income settings where the burden of both conditions is rising. Evidence from the D:A:D observational study suggests that increases in predicted cardiovascular and renal risk were associated with higher disease rates, further emphasizing the need for comprehensive risk assessment in routine HIV care [66, 67].

This analysis should be interpreted with caution for some reasons: (1) These groups likely differ at baseline in ways that independently influence outcomes, including disease stage, duration of infection and access to healthcare. Most included studies did not adequately adjust for such confounders. Accordingly, observed differences between subgroups may reflect residual confounding rather than true treatment effects. (2) Studies differed in design, ART‐exposure definitions and diagnosis criteria for metabolic outcomes. Metabolic syndrome, for example, was reported by at least using three criteria, which make direct comparisons challenging. (3) Using GRADE, we rated the overall evidence as low or very low because of study bias, inconsistent effect sizes, small subgroup samples and publication bias cannot be reliably confirmed or ruled out. (4) ART exposure was inconsistently defined across studies, with some reporting treatment status only ‘ART’ or ‘HAART’ without information on regimen composition, duration or cumulative exposure. To address this, regimen‐specific analyses were limited to studies with adequate exposure data. (5) We indexed temporal trends by publication year because enrolment dates were inconsistently reported and often spanned several years. Accordingly, our temporal analysis reflects the literature timeline rather than exact exposure timing; and (6) The review included studies from diverse global settings, but none of the studies were conducted in North America. While this may limit generalizability to regions with different ART prescribing practices, healthcare systems and baseline metabolic risk, NA‐ACCORD in North America found a similar excess metabolic risk when first‐line INSTIs were compared with NNRTI backbones after adjustment for weight gain [65].

## CONCLUSIONS

We observed evolving ART drug‐class patterns over time, though these temporal trends are constrained by the use of publication year as a proxy for treatment era and should be interpreted as reflecting the literature timeline rather than changes in clinical practice. ART‐treated adult people living with HIV have significantly higher odds of developing metabolic syndrome compared with those not yet receiving ART. However, as these groups likely differ in baseline characteristics such as disease stage, duration of infection and healthcare access, this association should be interpreted with caution. In contrast, our meta‐analytic evidence did not demonstrate a statistically significant association between ART use and the odds of developing type 2 diabetes mellitus. Substantial heterogeneity in study design, age distribution, ART exposure and temporal differences in treatment practices, combined with the potential for residual confounding in comparisons between ART‐treated and ART‐naïve populations, limits causal interpretation. These findings should therefore be understood as reflecting epidemiological patterns across historical treatment contexts rather than isolated effects of ART or specific drug classes. High quality, longer‐term prospective cohorts with standardized assessment that capture long‐term metabolic outcomes and adequate confounder adjustment are needed to refine risk–benefit assessment of modern ART and to inform personalized treatment and monitoring strategies.

## AUTHOR CONTRIBUTIONS

**Melani Ratih Mahanani:** Conceptualization; funding acquisition; data curation; formal analysis; investigation; methodology; project administration; resources; software; supervision; validation; visualization; writing—original draft preparation; writing—reviewing and editing. **Myo Chit:** Data curation; investigation; software; writing—original draft preparation; writing—reviewing and editing. **Sophie Mohr:** Data curation; investigation; software; writing—reviewing and editing. **Elisenda Cama i Gibernau:** Data curation; investigation; writing—reviewing and editing. **Svetlana Hetjens:** Methodology; validation; writing—reviewing and editing. **Volker Winkler:** Conceptualization; formal analysis; methodology; supervision; validation; writing—reviewing and editing. **Hans‐Michael Steffen:** Conceptualization; supervision; methodology; validation; writing—reviewing and editing. **Florian Neuhann:** Conceptualization; supervision; methodology; validation; writing—reviewing and editing. All authors have read and agreed to the published version of the manuscript.

## CONFLICT OF INTEREST STATEMENT

The authors declare no conflicts of interest.

## Supporting information


**Table S1.** Full search strategy.
**Table S2.** PRISMA checklist.
**Table S3.** GRADE certainty of evidence assessment, for studies included in the meta‐analysis and categorized by the study outcome.
**Table S4.** List of studies excluded at full‐text screening stage, with brief reasons and sorted by author.
**Figure S1.** Sensitivity analysis for metabolic syndrome outcome excluding studies with designs other than cross‐sectional. ART, antiretroviral therapy; IDF, International Diabetes Federation criteria; NCEP ATP III, National Cholesterol Education Program Adult Treatment Panel III criteria.
**Figure S2.** Sensitivity analysis for type 2 diabetes mellitus outcome excluding studies with designs other than cross‐sectional. ART, antiretroviral therapy.
**Figure S3.** ROBINS‐I v2 risk of bias assessment of all included studies.
**Figure S4.** ROBINS‐I v2 risk of bias assessment of all included studies (summary).

## Data Availability

All data used in this systematic review and meta‐analysis were extracted from published studies that are publicly available in bibliographic databases. The dataset underlying the analyses is available from the corresponding author upon reasonable request. No individual participant data were collected or generated for this review.
