# Supplementary material for: Temporal shifts in antiretroviral therapy regimens and metabolic outcomes in people living with HIV: A systematic review and meta‐analysis
Source: HIV Med. 2026 May 21;27(9):1399–420. doi: 10.1111/hiv.70260 (PMC13547439; doi:10.1111/hiv.70260)
Supplement: Supplementary file 1 [file HIV-27-1399-s001.docx]

**SUPPLEMENTARY MATERIAL**

**Temporal shifts in antiretroviral therapy regimens and metabolic outcomes in people living with HIV: a systematic review and meta-analysis**

[**Table 1.** Full search strategy	2](#_Toc209783288)

[**Table 2.** PRISMA checklist	4](#_Toc209783289)

[**Table 3.** GRADE certainty of evidence assessment, for studies included in the meta-analysis and categorized by the study outcome	8](#_Toc209783290)

[**Table 4.** List of studies excluded at full-text screening stage, with brief reasons and sorted by author	9](#_Toc209783291)

[**Figure 1.** Sensitivity analysis for metabolic syndrome outcome excluding studies with designs other than cross-sectional. Abbreviations: ART, antiretroviral therapy; IDF, International Diabetes Federation criteria; NCEP ATP III, National Cholesterol Education Program Adult Treatment Panel III criteria	6](#_Toc220336091)

[**Figure 2.** Sensitivity analysis for type 2 diabetes mellitus outcome excluding studies with designs other than cross-sectional. Abbreviations: ART, antiretroviral therapy.	6](#_Toc220336092)

[**Figure 3.** ROBINS-I v2 risk of bias assessment of all included studies	7](#_Toc220336093)

[**Figure 4.** ROBINS-I v2 risk of bias assessment of all included studies (summary)	8](#_Toc220336094)

**Table 1.** Full search strategy

| **Database** | **Search string** |
| --- | --- |
| PubMed | ((Anti-HIV Agent [MeSH] OR Anti-HIV drug [MeSH] OR AIDS drug [MeSH] OR anti-retroviral agent* [MeSH] OR CCR5 Receptor Antagonist* [MeSH] OR HIV Fusion Inhibitor* [MeSH] OR HIV Integrase Inhibitor* [MeSH] OR HIV Protease Inhibitor* [MeSH] OR nucleoside reverse transcriptase inhibitor [Title/Abstract] OR abacavir [Title/Abstract] OR zidovudine [Title/Abstract] OR lamivudine [Title/Abstract] OR stavudine [Title/Abstract] OR tenofovir disoproxil fumarate [Title/Abstract] OR tenofovir DF [Title/Abstract] OR tenofovir alafenamide [Title/Abstract] OR tenofovir AF [Title/Abstract] OR didanosine [Title/Abstract] OR emtricitabine [Title/Abstract] OR non-nucleoside reverse transcriptase inhibitor [Title/Abstract] OR nevirapine [Title/Abstract] OR efavirenz [Title/Abstract] OR etravirine [Title/Abstract] OR rilpivirine [Title/Abstract] OR protease inhibitor [Title/Abstract] OR lopinavir [Title/Abstract] OR ritonavir [Title/Abstract] OR atazanavir [Title/Abstract] OR darunavir [Title/Abstract] OR indinavir [Title/Abstract] OR nelfinavir [Title/Abstract] OR saquinavir [Title/Abstract] OR integrase inhibitor [Title/Abstract] OR dolutegravir [Title/Abstract] OR raltegravir [Title/Abstract]) AND (Diabetes Mellitus, Type 2 [MeSH] OR Diabetes Mellitus, Non-Insulin Dependent [Title/Abstract] OR Diabetes Mellitus, Type II [Title/Abstract] OR Type 2 Diabetes [Title/Abstract] OR Diabetes Mellitus, Adult Onset [Title/Abstract] OR Glucose Intolerance [MeSH] OR Glucose Intolerance [Title/Abstract] OR Impaired Glucose Tolerance* [Title/Abstract] OR dyslipidemias [MeSH] OR dyslipidemi* [Title/Abtract] OR dyslipoproteinemia [Title/Abtract] OR hypercholesterolemia [MeSH] OR metabolic syndrome [MeSH] OR insulin resistance syndrome [Title/Abstract] OR adiposity [MeSH] OR obesity [Title/Abstract] OR hypertension [MeSH] OR blood pressure, high [Title/Abstract] OR high blood pressure [Title/Abstract] OR cardiovascular diseases [MeSH] OR cardiac event [Title/Abstract] OR lipodystrophy [MeSH] OR lipodystroph* [Title/Abstract])) |
| CINAHL and Academic Search Complete via EBSCOHost | TI (( Anti-HIV Agent OR Anti-HIV drug OR AIDS drug OR anti-retroviral agent* OR CCR5 Receptor Antagonist* OR antiretroviral therapy OR antiretroviral treatment OR HIV Fusion Inhibitor* OR HIV Integrase Inhibitor* OR HIV Protease Inhibitor* OR nucleoside reverse transcriptase inhibitor OR abacavir OR zidovudine OR lamivudine OR stavudine OR tenofovir disoproxil fumarate OR tenofovir DF OR tenofovir alafenamide OR tenofovir AF OR didanosine OR emtricitabine OR non-nucleoside reverse transcriptase inhibitor OR nevirapine OR efavirenz OR etravirine OR rilpivirine OR protease inhibitor OR lopinavir OR ritonavir OR atazanavir OR darunavir OR indinavir OR nelfinavir OR saquinavir OR integrase inhibitor OR dolutegravir OR raltegravir ) AND ( Diabetes Mellitus, Type 2 OR Diabetes Mellitus, Non-Insulin Dependent OR Diabetes Mellitus, Type II OR Type 2 Diabetes OR Diabetes Mellitus, Adult Onset OR Glucose Intolerance OR Impaired Glucose Tolerance* OR dyslipidemi* OR dyslipoproteinemia OR hypercholesterolemia OR metabolic syndrome OR insulin resistance syndrome adiposity OR obesity OR hypertension OR blood pressure, high OR high blood pressure OR cardiovascular diseases OR cardiac event OR lipodystroph*)) |
| Web of Science | Anti-HIV Agent OR Anti-HIV drug OR AIDS drug OR anti-retroviral agent* OR CCR5 Receptor Antagonist* OR HIV Fusion Inhibitor* OR HIV Integrase Inhibitor* OR HIV Protease Inhibitor* OR nucleoside reverse transcriptase inhibitor OR abacavir OR zidovudine OR lamivudine OR stavudine OR tenofovir disoproxil fumarate OR tenofovir DF OR tenofovir alafenamide OR tenofovir AF OR didanosine OR emtricitabine OR non-nucleoside reverse transcriptase inhibitor OR nevirapine OR efavirenz OR etravirine OR rilpivirine OR protease inhibitor OR lopinavir OR ritonavir OR atazanavir OR darunavir OR indinavir OR nelfinavir OR saquinavir OR integrase inhibitor OR dolutegravir OR raltegravir (Title) and Diabetes Mellitus, Type 2 OR Diabetes Mellitus, Non-Insulin Dependent OR Diabetes Mellitus, Type II OR Type 2 Diabetes OR Diabetes Mellitus, Adult Onset OR Glucose Intolerance OR Impaired Glucose Tolerance* OR dyslipidemi* OR dyslipoproteinemia OR hypercholesterolemia OR metabolic syndrome OR insulin resistance syndrome adiposity OR obesity OR hypertension OR blood pressure, high OR high blood pressure OR cardiovascular diseases OR cardiac event OR lipodystroph* (Title) |
| Proquest | title(Anti-HIV Agent OR Anti-HIV drug OR AIDS drug OR anti-retroviral agent* OR CCR5 Receptor Antagonist* OR antiretroviral therapy OR antiretroviral treatment OR HIV Fusion Inhibitor* OR HIV Integrase Inhibitor* OR HIV Protease Inhibitor* OR nucleoside reverse transcriptase inhibitor OR abacavir OR zidovudine OR lamivudine OR stavudine OR tenofovir disoproxil fumarate OR tenofovir DF OR tenofovir alafenamide OR tenofovir AF OR didanosine OR emtricitabine OR non-nucleoside reverse transcriptase inhibitor OR nevirapine OR efavirenz OR etravirine OR rilpivirine OR protease inhibitor OR lopinavir OR ritonavir OR atazanavir OR darunavir OR indinavir OR nelfinavir OR saquinavir OR integrase inhibitor OR dolutegravir OR raltegravir) AND title(Diabetes Mellitus, Type 2 OR Diabetes Mellitus, Non-Insulin Dependent OR Diabetes Mellitus, Type II OR Type 2 Diabetes OR Diabetes Mellitus, Adult Onset OR Glucose Intolerance OR Impaired Glucose Tolerance* OR dyslipidemi* OR dyslipoproteinemia OR hypercholesterolemia OR metabolic syndrome OR insulin resistance syndrome adiposity OR obesity OR hypertension OR blood pressure, high OR high blood pressure OR cardiovascular diseases OR cardiac event OR lipodystroph*) |
| WHO Library | ti,wrdl: Anti-HIV Agent OR Anti-HIV drug OR AIDS drug OR anti-retroviral agent* OR CCR5 Receptor Antagonist* OR HIV Fusion Inhibitor* OR HIV Integrase Inhibitor* OR HIV Protease Inhibitor* OR nucleoside reverse transcriptase inhibitor OR abacavir OR zidovudine OR lamivudine OR stavudine OR tenofovir disoproxil fumarate OR tenofovir DF OR tenofovir alafenamide OR tenofovir AF OR didanosine OR emtricitabine OR non-nucleoside reverse transcriptase inhibitor OR nevirapine OR efavirenz OR etravirine OR rilpivirine OR protease inhibitor OR lopinavir OR ritonavir OR atazanavir OR darunavir OR indinavir OR nelfinavir OR saquinavir OR integrase inhibitor OR dolutegravir OR raltegravir AND ti,wrdl: Diabetes Mellitus, Type 2 OR Diabetes Mellitus, Non-Insulin Dependent OR Diabetes Mellitus, Type II OR Type 2 Diabetes OR Diabetes Mellitus, Adult Onset OR Glucose Intolerance OR Impaired Glucose Tolerance* OR dyslipidemi* OR dyslipoproteinemia OR hypercholesterolemia OR metabolic syndrome OR insulin resistance syndrome adiposity OR obesity OR hypertension OR blood pressure, high OR high blood pressure OR cardiovascular diseases OR cardiac event OR lipodystroph* |
| Global Index Medicus | Anti-HIV Agent OR Anti-HIV drug OR AIDS drug OR anti-retroviral agent* OR CCR5 Receptor Antagonist* OR HIV Fusion Inhibitor* OR HIV Integrase Inhibitor* OR HIV Protease Inhibitor* OR nucleoside reverse transcriptase inhibitor OR abacavir OR zidovudine OR lamivudine OR stavudine OR tenofovir disoproxil fumarate OR tenofovir DF OR tenofovir alafenamide OR tenofovir AF OR didanosine OR emtricitabine OR non-nucleoside reverse transcriptase inhibitor OR nevirapine OR efavirenz OR etravirine OR rilpivirine OR protease inhibitor OR lopinavir OR ritonavir OR atazanavir OR darunavir OR indinavir OR nelfinavir OR saquinavir OR integrase inhibitor OR dolutegravir OR raltegravir AND Diabetes Mellitus, Type 2 OR Diabetes Mellitus, Non-Insulin Dependent OR Diabetes Mellitus, Type II OR Type 2 Diabetes OR Diabetes Mellitus, Adult Onset OR Glucose Intolerance OR Impaired Glucose Tolerance* OR dyslipidemi* OR dyslipoproteinemia OR hypercholesterolemia OR metabolic syndrome OR insulin resistance syndrome adiposity OR obesity OR hypertension OR blood pressure, high OR high blood pressure OR cardiovascular diseases OR cardiac event OR lipodystroph* |

**Table 2.** PRISMA checklist

| **Section and Topic** | **Item #** | **Checklist item** | **Location where item is reported** |  |  |
| --- | --- | --- | --- | --- | --- |
| **TITLE** | | |  |  |  |
| Title | 1 | Identify the report as a systematic review. | 1 |  |  |
| **ABSTRACT** | | |  |  |  |
| Abstract | 2 | See the PRISMA 2020 for Abstracts checklist. | 2-3 |  |  |
| **INTRODUCTION** | | |  |  |  |
| Rationale | 3 | Describe the rationale for the review in the context of existing knowledge. | 3-4 |  |  |
| Objectives | 4 | Provide an explicit statement of the objective(s) or question(s) the review addresses. | 4 |  |  |
| **METHODS** | | |  |  |  |
| Eligibility criteria | 5 | Specify the inclusion and exclusion criteria for the review and how studies were grouped for the syntheses. | 5 |  |  |
| Information sources | 6 | Specify all databases, registers, websites, organisations, reference lists and other sources searched or consulted to identify studies. Specify the date when each source was last searched or consulted. | 5 |  |  |
| Search strategy | 7 | Present the full search strategies for all databases, registers and websites, including any filters and limits used. | 5, Supp. Table. 2 |  |  |
| Selection process | 8 | Specify the methods used to decide whether a study met the inclusion criteria of the review, including how many reviewers screened each record and each report retrieved, whether they worked independently, and if applicable, details of automation tools used in the process. | 6 |  |  |
| Data collection process | 9 | Specify the methods used to collect data from reports, including how many reviewers collected data from each report, whether they worked independently, any processes for obtaining or confirming data from study investigators, and if applicable, details of automation tools used in the process. | 6 |  |  |
| Data items | 10a | List and define all outcomes for which data were sought. Specify whether all results that were compatible with each outcome domain in each study were sought (e.g. for all measures, time points, analyses), and if not, the methods used to decide which results to collect. | 6 |  |  |
|  | 10b | List and define all other variables for which data were sought (e.g. participant and intervention characteristics, funding sources). Describe any assumptions made about any missing or unclear information. | 6 |  |  |
| Study risk of bias assessment | 11 | Specify the methods used to assess risk of bias in the included studies, including details of the tool(s) used, how many reviewers assessed each study and whether they worked independently, and if applicable, details of automation tools used in the process. | 6 |  |  |
| Effect measures | 12 | Specify for each outcome the effect measure(s) (e.g. risk ratio, mean difference) used in the synthesis or presentation of results. | 7 |  |  |
| Synthesis methods | 13a | Describe the processes used to decide which studies were eligible for each synthesis (e.g. tabulating the study intervention characteristics and comparing against the planned groups for each synthesis (item #5)). | 7 |  |  |
|  | 13b | Describe any methods required to prepare the data for presentation or synthesis, such as handling of missing summary statistics, or data conversions. | 7 |  |  |
|  | 13c | Describe any methods used to tabulate or visually display results of individual studies and syntheses. | 7 |  |  |
|  | 13d | Describe any methods used to synthesize results and provide a rationale for the choice(s). If meta-analysis was performed, describe the model(s), method(s) to identify the presence and extent of statistical heterogeneity, and software package(s) used. | 7 |  |  |
|  | 13e | Describe any methods used to explore possible causes of heterogeneity among study results (e.g. subgroup analysis, meta-regression). | 7 |  |  |
|  | 13f | Describe any sensitivity analyses conducted to assess robustness of the synthesized results. | 7 |  |  |
| Reporting bias assessment | 14 | Describe any methods used to assess risk of bias due to missing results in a synthesis (arising from reporting biases). | 7 |  |  |
| Certainty assessment | 15 | Describe any methods used to assess certainty (or confidence) in the body of evidence for an outcome. | 7 |  |  |
| **RESULTS** | | |  |  |  |
| Study selection | 16a | Describe the results of the search and selection process, from the number of records identified in the search to the number of studies included in the review, ideally using a flow diagram. | 7-8, Fig. 1 |  |  |
|  | 16b | Cite studies that might appear to meet the inclusion criteria, but which were excluded, and explain why they were excluded. | Supp. Table 4 |  |  |
| Study characteristics | 17 | Cite each included study and present its characteristics. | 8-18, Tab. 1 |  |  |
| Risk of bias in studies | 18 | Present assessments of risk of bias for each included study. | Supp. Figure 3, Figure 4 |  |  |
| Results of individual studies | 19 | For all outcomes, present, for each study: (a) summary statistics for each group (where appropriate) and (b) an effect estimate and its precision (e.g. confidence/credible interval), ideally using structured tables or plots. | 19-20, Figure 5, Figure 6 |  |  |
| Results of syntheses | 20a | For each synthesis, briefly summarise the characteristics and risk of bias among contributing studies. | 20,21 |  |  |
|  | 20b | Present results of all statistical syntheses conducted. If meta-analysis was done, present for each the summary estimate and its precision (e.g. confidence/credible interval) and measures of statistical heterogeneity. If comparing groups, describe the direction of the effect. | 19-20, Figure 5, Figure 6 |  |  |
|  | 20c | Present results of all investigations of possible causes of heterogeneity among study results. | 19-20, Figure 5, Figure 6 |  |  |
|  | 20d | Present results of all sensitivity analyses conducted to assess the robustness of the synthesized results. | 21, Supp. Figure 2, Figure 3 |  |  |
| Reporting biases | 21 | Present assessments of risk of bias due to missing results (arising from reporting biases) for each synthesis assessed. | Supp. Figure 3, Figure 4 |  |  |
| Certainty of evidence | 22 | Present assessments of certainty (or confidence) in the body of evidence for each outcome assessed. | Supp. Table 3 |  |  |
| **DISCUSSION** | | |  |  |  |
| Discussion | 23a | Provide a general interpretation of the results in the context of other evidence. | 22 |  |  |
|  | 23b | Discuss any limitations of the evidence included in the review. | 22 - 23 |  |  |
|  | 23c | Discuss any limitations of the review processes used. | 22 - 23 |  |  |
|  | 23d | Discuss implications of the results for practice, policy, and future research. | 23 |  |  |
| **OTHER INFORMATION** | | |  |  |  |
| Registration and protocol | 24a | Provide registration information for the review, including register name and registration number, or state that the review was not registered. | 5 |  |  |
|  | 24b | Indicate where the review protocol can be accessed, or state that a protocol was not prepared. | 5 |  |  |
|  | 24c | Describe and explain any amendments to information provided at registration or in the protocol. | 21 |  |  |
| Support | 25 | Describe sources of financial or non-financial support for the review, and the role of the funders or sponsors in the review. | 24 |  |  |
| Competing interests | 26 | Declare any competing interests of review authors. | 24 |  |  |
| Availability of data, code and other materials | 27 | Report which of the following are publicly available and where they can be found: template data collection forms; data extracted from included studies; data used for all analyses; analytic code; any other materials used in the review. | 24 |  |  |

**Figure 1.** Sensitivity analysis for metabolic syndrome outcome excluding studies with designs other than cross-sectional. Abbreviations: ART, antiretroviral therapy; IDF, International Diabetes Federation criteria; NCEP ATP III, National Cholesterol Education Program Adult Treatment Panel III criteria

**Figure 2.** Sensitivity analysis for type 2 diabetes mellitus outcome excluding studies with designs other than cross-sectional. Abbreviations: ART, antiretroviral therapy.

**Figure 3.** ROBINS-I v2 risk of bias assessment of all included studies

**Figure 4.** ROBINS-I v2 risk of bias assessment of all included studies (summary)

**Table 3.** GRADE certainty of evidence assessment, for studies included in the meta-analysis and categorized by the study outcome

| **People:** Adults living with HIV  **Settings:** Global population  **Intervention:** ART  **Comparison:** No-ART (Naïve) | | | | |
| --- | --- | --- | --- | --- |
| **Outcomes** | **Number of participants (studies)** | **Relative effect**  **(95% CI)** | **Study designs** | **Certainty of the evidence (GRADE)^†^** |
| Metabolic syndrome  *All combination, all diagnosis criteria* | 2,399 (7) | 2.16 (1.33 – 3.52) | 1 case control, 5 cross-sectional | ⊕⊕⊖⊖  Low |
| Type 2 diabetes mellitus *Intervention: 2 NRTIs + 1 NNRTI* | 1,888 (5) | 1.37 (0.65 – 2.90) | 1 retrospective cohort, 4 cross-sectional | ⊕⊕⊖⊖ Low |
| 95% CI: 95% Confidence interval  **^†^** GRADE Working Group grades of evidence  **Low** = This research provides some indication of the likely effect. However, the likelihood that it will be substantially different^‡^ is high.  ^‡^ Substantially different = a large enough difference that it might affect a decision | | | | |

**Table 4.** List of studies excluded at full-text screening stage, with brief reasons and sorted by author

| **Author** | **Year** | **Study Title** | **Reason for Exclusion** |
| --- | --- | --- | --- |
| Abah et al. | 2015 | Incidence and predictors of adverse drug events in an African cohort of HIV-infected adults treated with efavirenz | Did not include relevant comparator |
| Abdela et al. | 2023 | Prevalence and risk factors of metabolic syndrome in Ethiopia: describing an emerging outbreak in HIV clinics of the sub-Saharan Africa - a cross-sectional study | Did not include relevant comparator |
| Abebe et al. | 2014 | Antiretroviral treatment associated hyperglycemia and dyslipidemia among HIV infected patients at Burayu Health Center, Addis Ababa, Ethiopia: a cross-sectional comparative study | Did not report relevant outcomes (metabolic measures) |
| Aberash et al. | 2022 | Could a Dolutegravir-Based Antiretroviral Therapy Lead to Clinical Obesity? A Retrospective Cohort Study Conducted at Hawassa University Comprehensive Specialized Hospital in Hawassa, Sidama, Ethiopia | Did not include relevant comparator |
| Aberg et al. | 2017 | Pitavastatin versus pravastatin in adults with HIV-1 infection and dyslipidaemia (INTREPID): 12 week and 52-week results of a phase 4, multicentre, randomised, double-blind, superiority trial | Intervention not relevant to review question |
| Abrahams et al. | 2015 | Changes in blood pressure, glucose levels, insulin secretion and anthropometry after long term exposure to antiretroviral therapy in South African women | Did not include relevant comparator |
| Abrahams et al. | 2018 | Anthropometric definitions for antiretroviral-associated lipodystrophy derived from a longitudinal South African cohort with serial dual-energy X-ray absorptiometry measurements | Did not include relevant comparator |
| Achhra et al. | 2016 | Short-term weight gain after antiretroviral therapy initiation and subsequent risk of cardiovascular disease and diabetes: the D:A:D study | Did not include relevant comparator |
| Achwoka et al. | 2020 | High prevalence of non-communicable diseases among key populations enrolled at a large HIV prevention & treatment program in Kenya | Did not assess relevant exposures |
| Adébayo et al. | 2015 | Prevalence, associated and predisposing factors of metabolic syndrome among people living with HIV on antiretroviral treatment in Porto Novo in 2014 | Study not available in English |
| Adewole et al. | 2010 | Lipid profile in HIV/AIDS patients in Nigeria | Did not include relevant comparator |
| Agbeko et al. | 2019 | Patients living with human immunodeficiency virus on antiretroviral therapy diagnosed with lipid and carbohydrate disorders who are at risk of cardio-vascular disease: study conducted at the Medical Centre of the ONG Espoir Vie in Lomé | Did not include relevant comparator |
| Albuquerque et al. | 2005 | High frequency of Fredrickson's phenotypes IV and IIb in Brazilians infected by human immunodeficiency virus | Did not include relevant comparator |
| Alikhani et al. | 2019 | Association between lipodystrophy and length of exposure to ARTs in adult HIV-1 infected patients in Montreal | Did not include relevant comparator |
| Alvi et al. | 2018 | Protease Inhibitors and Cardiovascular Outcomes in Patients With HIV and Heart Failure | Did not include relevant comparator |
| Amare et al. | 2021 | Predictors of glucose metabolism and blood pressure among Ethiopian individuals with HIV/AIDS after one-year of antiretroviral therapy | Did not include relevant comparator |
| Anastos et al. | 2007 | Association of Serum Lipid Levels With HIV Serostatus, Specific Antiretroviral Agents, and Treatment Regimens | Did not report relevant outcomes (metabolic measures) |
| Andany et al. | 2011 | Ethnicity and gender differences in lipodystrophy of HIV-positive individuals taking antiretroviral therapy in Ontario, Canada | Did not include relevant comparator |
| Antoniou et al. | 2004 | A comparison of dyslipidemias associated with either lopinavir/ritonavir- or indinavir/ritonavir-based antiretroviral therapy | Not an eligible publication type |
| Assogba et al. | 2024 | Prevalence and associated factors of hypertension among people living with HIV/AIDS on antiretroviral therapy in Benin's National Teaching Hospital | Did not report relevant outcomes |
| Asundi et al. | 2022 | Integrase Inhibitor Use Associated with Weight Gain in Women and Incident Diabetes Mellitus | Did not include relevant comparator |
| Bahamdain | 2022 | Effect of Dolutegravir on Plasma Glucose Among Human Immunodeficiency Virus Patients in a Community Health Center Setting | Did not include relevant comparator |
| Baker et al. | 2017 | Changes in Cardiovascular Disease Risk Factors With Immediate Versus Deferred Antiretroviral Therapy Initiation Among HIV-Positive Participants in the START (Strategic Timing of Antiretroviral Treatment) Trial | Did not include relevant comparator |
| Bam et al. | 2020 | The association between HIV tri-therapy with the development of Type-2 Diabetes Mellitus in a rural South African District: A case-control study | Did not include relevant comparator |
| Bannister et al. | 2022 | Changes in body mass index and clinical outcomes after initiation of contemporary antiretroviral regimens | Did not include relevant comparator |
| Barbaro et al. | 2003 | An open-label, prospective, observational study of the incidence of coronary artery disease in patients with HIV infection receiving highly active antiretroviral therapy | Did not include relevant comparator |
| Batavia et al. | 2018 | Blood pressure and mortality in a prospective cohort of HIV-infected adults in Port-au-Prince, Haiti | Did not include relevant comparator |
| Batte et al. | 2024 | Health-related quality of life and hypertension in people with HIV on long-term antiretroviral therapy in Uganda | Did not report relevant outcomes |
| Behrens et al. | 1999 | Impaired glucose tolerance, beta cell function and lipid metabolism in HIV patients under treatment with protease inhibitors | Did not include relevant comparator |
| Bellinati et al. | 2020 | Association of Lower Adiponectin Plasma Levels, Increased Age and Smoking with Subclinical Atherosclerosis in Patients with HIV-1 Infection | Unable to retrieve full text |
| Bergersen et al. | 2005 | Low prevalence of high-density lipoprotein cholesterol level < 1 mmol/L in non-nucleoside reverse transcriptase inhibitor recipients | Did not include relevant comparator |
| Bernal et al. | 2007 | Insulin resistance in HIV-infected patients receiving long-term therapy with efavirenz, lopinavir/ritonavir and atazanavir | Study not available in English |
| Bernal Morell et al. | 2016 | The CD4/CD8 Ratio is Inversely Associated with Carotid Intima-Media Thickness Progression in Human Immunodeficiency Virus-Infected Patients on Antiretroviral Treatment | Did not include relevant comparator |
| Biron et al. | 2012 | Metabolic syndrome in French HIV-infected patients: prevalence and predictive factors after 3 years of antiretroviral therapy | Did not include relevant comparator |
| Bleasel et al. | 2020 | Body mass index and noninfectious comorbidity in HIV-positive patients commencing antiretroviral therapy in Zimbabwe | Did not include relevant comparator |
| Blümer et al. | 2008 | Zidovudine/lamivudine contributes to insulin resistance within 3 months of starting combination antiretroviral therapy | Did not include relevant comparator |
| Bogner et al. | 2001 | Stavudine versus zidovudine and the development of lipodystrophy | Did not include relevant comparator |
| Bollens et al. | 2004 | Major hypertriglyceridemia in HIV-infected patients on antiretroviral therapy: a role of the personal and family history | Did not include relevant comparator |
| Bongiovanni et al. | 2008 | Predictive factors of vascular intima media thickness in HIV-positive subjects | Did not report relevant outcomes (cardiovascular outcome) |
| Bonjoch et al. | 2013 | Switching the third drug of antiretroviral therapy to maraviroc in aviraemic subjects: a pilot, prospective, randomized clinical trial | Did not include relevant comparator |
| Boothby et al. | 2009 | Adipocyte differentiation, mitochondrial gene expression and fat distribution: differences between zidovudine and tenofovir after 6 months | Did not include relevant comparator |
| Botha et al. | 2014 | Cardiometabolic Changes in Treated Versus Never Treated HIV-Infected Black South Africans: The PURE Study | Did not report relevant outcomes (metabolic measures) |
| Bourgi et al. | 2020 | Weight gain among treatment-naive persons with HIV starting integrase inhibitors compared to non-nucleoside reverse transcriptase inhibitors or protease inhibitors in a large observational cohort in the United States and Canada | Did not include relevant comparator |
| Boyd et al. | 2017 | Body composition and metabolic outcomes after 96 weeks of treatment with ritonavir-boosted lopinavir plus either nucleoside or nucleotide reverse transcriptase inhibitors or raltegravir in patients with HIV with virological failure of a standard first-line antiretroviral therapy regimen: a substudy of the randomised, open-label, non-inferiority SECOND-LINE study | Did not include relevant comparator |
| Bozzette et al. | 2003 | Cardiovascular and cerebrovascular events in patients treated for human immunodeficiency virus infection | Did not include relevant comparator |
| Brennan et al. | 2018 | Prevalence, incidence, predictors, treatment, and control of hypertension among HIV-positive adults on antiretroviral treatment in public sector treatment programs in South Africa | Did not include relevant comparator |
| Brown et al. | 2010 | Association between systemic inflammation and incident diabetes in HIV-infected patients after initiation of antiretroviral therapy | Did not include relevant comparator |
| Brunet et al. | 2021 | Switch from Tenofovir Disoproxil Fumarate to Tenofovir Alafenamide in People Living with HIV: Lipid Changes and Statin Underutilization | Did not include relevant comparator |
| Buchacz et al. | 2008 | Changes in lipid profile over 24 months among adults on first-line highly active antiretroviral therapy in the home-based AIDS care program in rural Uganda | Did not include relevant comparator |
| Bucher et al. | 2004 | Protease inhibitor-sparing simplified maintenance therapy: a need for perspective | Not an eligible publication type |
| Busti et al. | 2008 | Improvement in insulin sensitivity and dyslipidemia in protease inhibitor-treated adult male patients after switch to atazanavir/ritonavir | Did not include relevant comparator |
| Byereta et al. | 2024 | Prevalence and factors associated with hyperglycemia among persons living with HIV/AIDS on dolutegravir-based antiretroviral therapy in Uganda | Not using an eligible study design |
| Byonanebye et al. | 2024 | Associations between change in BMI and the risk of hypertension and dyslipidaemia in people receiving integrase strand-transfer inhibitors, tenofovir alafenamide, or both compared with other contemporary antiretroviral regimens: a multicentre, prospective observational study from the RESPOND consortium cohorts | Did not include relevant comparator |
| Byonanebye et al. | 2022 | Incidence of hypertension in people with HIV who are treated with integrase inhibitors versus other antiretroviral regimens in the RESPOND cohort consortium | Did not include relevant comparator |
| Byonanebye et al. | 2023 | Prevalence and incidence of hypertension in a heavily treatment-experienced cohort of people living with HIV in Uganda | Did not include relevant comparator |
| Cahn et al. | 2004 | Atazanavir‚ÄîA Once-daily HIV Protease Inhibitor That Does Not Cause Dyslipidemia in Newly Treated Patients: Results from Two Randomized Clinical Trials | Did not include relevant comparator |
| Calza et al. | 2005 | Substitution of nevirapine or efavirenz for protease inhibitor versus lipid-lowering therapy for the management of dyslipidaemia | Did not include relevant comparator |
| Calza et al. | 2003 | Incidence of hyperlipidaemia in a cohort of 212 HIV-infected patients receiving a protease inhibitor-based antiretroviral therapy | Did not include relevant comparator |
| Calza et al. | 2009 | Cardiovascular risk factors and ultrasound evaluation of carotid atherosclerosis in patients with HIV-1 infection | Did not report relevant outcomes |
| Caramelli et al. | 2001 | Hyperlipidemia related to the use of HIV-protease inhibitors: natural history and results of treatment with fenofibrate | Did not report relevant outcomes |
| Cardoso-Neto et al. | 2023 | Weight gain in patients starting Dolutegravir-based ART according to baseline CD4 count after 48 weeks of follow up | Did not include relevant comparator |
| Carr et al. | 2001 | HIV protease inhibitor substitution in patients with lipodystrophy: a randomized, controlled, open-label, multicentre study | Did not include relevant comparator |
| Carvalho et al. | 2018 | Evaluation of inflammatory biomarkers, carotid intima-media thickness and cardiovascular risk in HIV-1 treatment-naive patients | Research question not relevant to review |
| Ceccato et al. | 2011 | Antiretroviral therapy-associated dyslipidemia in patients from a reference center in Brazil | Did not include relevant comparator |
| Chen et al. | 2025 | Prevalence of common chronic diseases and related factors in HIV-infected persons in Henan Province, 2023 | Study not available in English |
| Chêne et al. | 2002 | Role of long-term nucleoside-analogue therapy in lipodystrophy and metabolic disorders in human immunodeficiency virus-infected patients | Did not include relevant comparator |
| Chow et al. | 2015 | Elevated Blood Pressure in HIV-Infected Individuals Receiving Highly Active Antiretroviral Therapy | Did not report relevant outcomes (cardiovascular measures) |
| Ciccullo et al. | 2025 | Cardiovascular Safety of Doravirine/Lamivudine/Tenofovir Disoproxil Fumarate in Virologically Suppressed PLWHIV: A Comparative Analysis of CVD Scores | Did not report relevant outcomes |
| Claas et al. | 2007 | Metabolic and anthropometric changes one year after switching from didanosine/stavudine to tenofovir in HIV-infected patients | Did not include relevant comparator |
| Cocohoba et al. | 2009 | The SWITCHMRK studies: substitution of lopinavir/ritonavir with raltegravir in HIV-positive individuals | Did not include relevant comparator |
| Colafigli et al. | 2008 | Cardiovascular risk score change in HIV-1-infected patients switched to an atazanavir-based combination antiretroviral regimen | Did not include relevant comparator |
| Corral et al. | 2009 | Cerebrovascular ischemic events in HIV-1-infected patients receiving highly active antiretroviral therapy: incidence and risk factors | Did not include relevant comparator |
| Crane et al. | 2006 | Antiretroviral medications associated with elevated blood pressure among patients receiving highly active antiretroviral therapy | Did not include relevant comparator |
| Dai et al. | 2019 | Impact of Lopinavir/Ritonavir and Efavirenz-Based Antiretroviral Therapy on the Lipid Profile of Chinese HIV/AIDS Treatment-Naive Patients in Beijing: A Retrospective Study | Unable to retrieve full text |
| Darnell et al. | 2021 | Impact of switching to tenofovir alafenamide on weight gain as compared to maintaining a non-tenofovir alafenamide containing regimen | Did not include relevant comparator |
| Dave et al. | 2016 | Anti-Retroviral Therapy Increases the Prevalence of Dyslipidemia in South African HIV-Infected Patients | Did not report relevant outcomes (metabolic measures) |
| de Saint-Martin et al. | 2010 | Impact of atazanavir-based HAART regimen on the carotid intima-media thickness of HIV-infected persons: a comparative prospective cohort | Did not report relevant outcomes |
| Dimala et al. | 2016 | Prevalence of Hypertension in HIV/AIDS Patients on Highly Active Antiretroviral Therapy (HAART) Compared with HAART Naïve Patients at the Limbe Regional Hospital, Cameroon | Did not report relevant outcomes (cardiovascular outcome) |
| Dobe et al. | 2020 | Patterns of cardiovascular risk and disease in HIV-positive adults on anti-retroviral therapy in Mozambique | Did not include relevant comparator |
| Domingo et al. | 2014 | A 48-week study of fat molecular alterations in HIV naive patients starting tenofovir/emtricitabine with lopinavir/ritonavir or efavirenz | Did not include relevant comparator |
| Domingos et al. | 2009 | Metabolic Effects Associated to the Highly Active Antiretroviral Therapy (HAART) in AIDS Patients | Did not report relevant outcomes (metabolic measures) |
| Dragoviƒá et al. | 2014 | Lipodystrophy induced by combination antiretroviral therapy in HIV/AIDS patients: A Belgrade cohort study | Did not include relevant comparator |
| Dubé et al. | 2005 | Glucose metabolism, lipid, and body fat changes in antiretroviral-naive subjects randomized to nelfinavir or efavirenz plus dual nucleosides | Did not include relevant comparator |
| Dubé et al. | 2010 | Relationship of Body Composition, Metabolic Status, Antiretroviral Use, and HIV Disease Factors to Endothelial Dysfunction in HIV-Infected Subjects | Did not report relevant outcomes (cardiovascular outcome) |
| Duro et al. | 2013 | Lipid profile changes by high activity anti-retroviral therapy | Not an eligible publication type |
| Dysangco et al. | 2017 | HIV infection, antiretroviral therapy, and measures of endothelial function, inflammation, metabolism, and oxidative stress | Did not report relevant outcomes |
| Dzudie et al. | 2021 | Hypertension among people living with HIV/AIDS in Cameroon: A cross-sectional analysis from Central Africa International Epidemiology Databases to Evaluate AIDS | Did not report relevant outcomes (cardiovascular outcome) |
| Ekun et al. | 2021 | Risks of cardio-vascular diseases among highly active antiretroviral therapy (HAART) treated HIV seropositive volunteers at a treatment centre in Lagos, Nigeria | Did not report relevant outcomes (cardiovascular outcome) |
| Elgalib et al. | 2011 | The assessment of metabolic syndrome in UK patients with HIV using two different definitions: CREATE 2 study | Did not include relevant comparator |
| Ena et al. | 2004 | Abnormal body fat distribution and type of antiretroviral therapy as predictors of cardiovascular disease risk in HIV-infected patients | Study not available in English |
| Erlandson et al. | 2021 | Weight Change Following Antiretroviral Therapy Switch in People With Viral Suppression: Pooled Data from Randomized Clinical Trials | Did not include relevant comparator |
| Estrada et al. | 2002 | Long-term metabolic consequences of switching from protease inhibitors to efavirenz in therapy for human immunodeficiency virus-infected patients with lipoatrophy | Did not include relevant comparator |
| Estrada et al. | 2011 | Dyslipidaemia in HIV-infected women on antiretroviral therapy. Analysis of 922 patients from the Spanish VACH cohort | Did not include relevant comparator |
| Estrada et al. | 2006 | Lipodystrophy and metabolic syndrome in HIV-infected patients treated with antiretroviral therapy | Did not include relevant comparator |
| Fan et al. | 2020 | Incidence of hypertension among persons living with HIV in China: a multicenter cohort study | Did not include relevant comparator |
| Fathey et al. | 2022 | Visceral Adipose Tissue-Derived Serine Protease Inhibitor (VASPIN) as A Prognostic Marker in Systemic Hypertension | Did not report relevant outcomes |
| Feleke et al. | 2012 | Prevalence of highly active antiretroviral therapy associated metabolic abnormalities and lipodystrophy in HIV infected patients | Unable to retrieve full text |
| Ferrer et al. | 2011 | Impact of switching from lopinavir/ritonavir to atazanavir/ritonavir on body fat redistribution in virologically suppressed HIV-infected adults | Did not include relevant comparator |
| Fisac et al. | 2005 | Metabolic benefits 24 months after replacing a protease inhibitor with abacavir, efavirenz or nevirapine | Did not include relevant comparator |
| Flexor et al. | 2013 | Aging and HIV infection: 4 years follow-up of 149 HIV infected patients older than 60 years in West Paris agglomeration (COREVIH Île-de-France Ouest) | Study not available in English |
| Friis-Møller et al. | 2003 | Cardiovascular disease risk factors in HIV patients--association with antiretroviral therapy. Results from the DAD study | Not an eligible publication type |
| Gagliardini et al. | 2017 | Lipid-lowering effect and changes in estimated cardiovascular risk after switching to a tenofovir-containing regimen for the treatment of HIV-infected patients | Did not include relevant comparator |
| Galli et al. | 2002 | Incidence of adipose tissue alterations in first-line antiretroviral therapy: the LipoICoNa Study | Did not include relevant comparator |
| Galli et al. | 2002 | Body habitus changes and metabolic alterations in protease inhibitor-naive HIV-1-infected patients treated with two nucleoside reverse transcriptase inhibitors | Did not include relevant comparator |
| Gan et al. | 2002 | Altered myocellular and abdominal fat partitioning predict disturbance in insulin action in HIV protease inhibitor-related lipodystrophy | Research question not relevant to review |
| García-Abellán et al. | 2025 | No accelerated progression of subclinical atherosclerosis with integrase strand transfer inhibitors compared to non-nucleoside reverse transcriptase inhibitors | Did not report relevant outcomes |
| García-Ruiz de Morales et al. | 2025 | Metabolic Complications After Initiating BIC/FTC/TAF Versus DTG + 3TC in ART-naive Adults With Human Immunodeficiency Virus (HIV): A Multicenter Prospective Cohort Study | Did not include relevant comparator |
| Gazzola et al. | 2021 | Dyslipidaemia after switch to tenofovir alafenamide (TAF)-based cART regimens in a cohort of HIV-positive patients: what clinical relevance? | Did not include relevant comparator |
| Geldres-Molina et al. | 2021 | Lipid profile levels in HIV-AIDS patients on treatment with efavirenz and atazanavir. Cohort study | Study not available in English |
| Gleason et al. | 2015 | Current Efavirenz (EFV) or Ritonavir-Boosted Lopinavir (LPV/r) Use Correlates with Elevate Markers of Atherosclerosis in HIV-Infected Subjects in Addis Ababa, Ethiopia | Did not report relevant outcomes (cardiovascular outcome) |
| Gleason et al. | 2016 | Efavirenz and ritonavir-boosted lopinavir use exhibited elevated markers of atherosclerosis across age groups in people living with HIV in Ethiopia | Did not report relevant outcomes (cardiovascular outcome) |
| Gomes et al. | 2016 | Incidence of Diabetes Mellitus and Obesity and the Overlap of Comorbidities in HIV+ Hispanics Initiating Antiretroviral Therapy | Did not include relevant comparator |
| Gómez-Ayerbe et al. | 2022 | Weight changes after first-line antiretroviral initiation in a cohort of HIV-positive patients in Southern Spain (CAPOTA study) | Did not include relevant comparator |
| Gonah et al. | 2020 | Prevalence of diabetes mellitus and hypertension in people living with human immunodeficiency virus on antiretroviral therapy in Gweru district, Zimbabwe | Did not include relevant comparator |
| González-Cordón et al. | 2018 | Subclinical cardiovascular disease in patients starting contemporary protease inhibitors | Did not include relevant comparator |
| González-Domenech et al. | 2021 | Synergic effect of metabolic syndrome and lipodystrophy on oxidative stress and inflammation process in treated HIV-patients | Did not assess relevant exposures |
| Gotti et al. | 2012 | Increase in standard cholesterol and large HDL particle subclasses in antiretroviral-naïve patients prescribed efavirenz compared to atazanavir/ritonavir | Did not include relevant comparator |
| Grandominico and Fichtenbaum | 2008 | Short-Term Effect of HAART on Blood Pressure in HIV-Infected Individuals | Did not report relevant outcomes (cardiovascular measures) |
| Grinspoon et al. | 2025 | Performance of the pooled cohort equations and D:A:D risk scores among individuals with HIV in a global cardiovascular disease prevention trial: a cohort study leveraging data from REPRIEVE | Not using an eligible study design |
| Grover et al. | 2005 | Impact of dyslipidemia associated with Highly Active Antiretroviral Therapy (HAART) on cardiovascular risk and life expectancy | Did not include relevant comparator |
| Guaraldi et al. | 2011 | Morphological and Metabolic Components of Lipodystrophy in Various Nevirapine-Based Highly Active Antiretroviral Therapy (HAART) Regimens | Did not include relevant comparator |
| Guehi et al. | 2016 | High prevalence of being Overweight and Obese HIV-infected persons, before and after 24 months on early ART in the ANRS 12136 Temprano Trial | Did not include relevant comparator |
| Guiguet et al. | 2008 | Influence of control selection in nested case-control studies: the example of exposure to antiretroviral treatment (ART) and the risk of myocardial infarction (MI) in the French Hospital Database on HIV (FHDH-ANRS CO4) | Did not include relevant comparator |
| Hamooya et al. | 2021 | Metabolic syndrome in Zambian adults with human immunodeficiency virus on antiretroviral therapy: Prevalence and associated factors | Did not include relevant comparator |
| Han et al. | 2009 | Prevalence of and clinical factors associated with lipoatrophy in HIV-infected Koreans receiving highly active antiretroviral therapy | Did not include relevant comparator |
| Han et al. | 2019 | Diabetes mellitus burden among people living with HIV from the Asia-Pacific region | Did not include relevant comparator |
| Hatleberg et al. | 2018 | Association between exposure to antiretroviral drugs and the incidence of hypertension in HIV-positive persons: the Data Collection on Adverse Events of Anti-HIV Drugs (D:A:D) study | Did not include relevant comparator |
| Haubrich et al. | 2009 | Metabolic outcomes in a randomized trial of nucleoside, nonnucleoside and protease inhibitor-sparing regimens for initial HIV treatment | Did not include relevant comparator |
| He et al. | 2020 | Widespread use of anti-retroviral therapy elevated the prevalence of dyslipidemia in HIV-infected Chinese patients | Did not assess relevant exposures |
| Heath et al. | 2002 | Incidence of morphological and lipid abnormalities: gender and treatment differentials after initiation of first antiretroviral therapy | Did not include relevant comparator |
| Hiransuthikul et al. | 2007 | Lipid profiles of Thai adult HIV-infected patients receiving protease inhibitors | Did not include relevant comparator |
| Hirigo et al. | 2023 | Effect of dolutegravir-based first-line antiretroviral therapy on weight and body mass index among adult people living with HIV on follow up at health facilities in Hawassa city administration, Southern Ethiopia: a retrospective cohort study | Did not include relevant comparator |
| Hirigo et al. | 2024 | The association between dolutegravir-based antiretrovirals and high blood pressure among adults with HIV in southern Ethiopia: a cross-sectional study | Did not report relevant outcomes |
| Høgh et al. | 2022 | Insulin resistance in people living with HIV is associated with exposure to thymidine analogues and/or didanosine and prior immunodeficiency | Did not include relevant comparator |
| Holroyd et al. | 2024 | Framingham risk score based vascular outcomes in acute versus chronic HIV cohorts after 6 years of ART | Did not include relevant comparator |
| Hosseini et al. | 2021 | Association between exposure to Efavirenz and substrates of dysrhythmia in HIV-infected young adults | Did not include relevant comparator |
| Hsu et al. | 2024 | Weight and metabolic changes among virally suppressed people with HIV who switched to co-formulated bictegravir/emtricitabine/tenofovir alafenamide | Did not include relevant comparator |
| Hsu et al. | 2021 | Incident type 2 diabetes mellitus after initiation of common HIV antiretroviral drugs | Did not include relevant comparator |
| Hsue et al. | 2024 | Risk of hypertension in treatment-naive people with HIV (PWH) in the US receiving integrase strand inhibitors (INSTIs) versus non-nucleoside reverse transcriptase inhibitors (NNRTIs) or tenofovir alafenamide (TAF) versus non-TAF-based regimens: pooled analysis of blood pressure data from five clinical trials | Did not report relevant outcomes |
| Iannone et al. | 2022 | Cardiovascular Disease Risk in a Cohort of Virologically Suppressed People Living with HIV Switching to Doravirine: Preliminary Data from the Real Life | Did not include relevant comparator |
| Iannone et al. | 2023 | Changes in Metabolic Profile in PLWHIV Switching to Doravirine-Based Regimen | Did not include relevant comparator |
| Isa et al. | 2017 | Hypertension in treated and untreated patients with HIV: a study from 2011 to 2013 at the Jos University Teaching Hospital, Nigeria | Did not assess relevant exposures |
| Iwuji et al. | 2010 | Abacavir is associated with increased risk of cardiovascular disease in HIV-infected patients: a UK clinic case-control study | Unable to retrieve full text |
| Januel et al. | 2019 | Brief Report: Impact of ART Classes on the Increasing Risk of Cerebral Small-Vessel Disease in Middle-Aged, Well-Controlled, cART-Treated, HIV-Infected Individuals | Not an eligible publication type |
| Jaschinski et al. | 2023 | Recent abacavir use and incident cardiovascular disease in contemporary-treated people with HIV | Not an eligible publication type |
| Jemal et al. | 2024 | Overweight and Obesity Among People Living With HIV on Dolutegravir- and Efavirenz-Based Therapies: A Comparative Cross-Sectional Study | Not using an eligible study design |
| Jemal et al. | 2023 | Blood glucose level and serum lipid profiles among people living with HIV on dolutegravir-based versus efavirenz-based cART; a comparative cross-sectional study | Did not include relevant comparator |
| Jemsek et al. | 2006 | Body fat and other metabolic effects of atazanavir and efavirenz, each administered in combination with zidovudine plus lamivudine, in antiretroviral-naive HIV-infected patients | Did not include relevant comparator |
| John et al. | 2003 | Randomized, controlled, 48-week study of switching stavudine and/or protease inhibitors to combivir/abacavir to prevent or reverse lipoatrophy in HIV-infected patients | Did not include relevant comparator |
| Joly et al. | 2002 | Increased risk of lipoatrophy under stavudine in HIV-1-infected patients: results of a substudy from a comparative trial | Did not include relevant comparator |
| Jong et al. | 2009 | The hemostatic balance in HIV-infected patients with and without antiretroviral therapy: partial restoration with antiretroviral therapy | Did not report relevant outcomes |
| Jose et al. | 2020 | Overweight and Obesity among Recipients of Antiretroviral Therapy at HIV Clinics in Gaborone, Botswana: Factors Associated with Change in Body Mass Index | Did not include relevant comparator |
| Juma et al. | 2019 | Cardiovascular risk factors among people living with HIV in rural Kenya: a clinic-based study | Did not report relevant outcomes (cardiovascular outcome) |
| Kalinjuma et al. | 2023 | Body mass index trends and its impact of under and overweight on outcome among PLHIV on antiretroviral treatment in rural Tanzania: A prospective cohort study | Did not include relevant comparator |
| Kaluba et al. | 2023 | Vascular dysfunction and body mass index in African adults with HIV | Did not include relevant comparator |
| Kamdem et al. | 2018 | Prevalence and risk factors of peripheral artery disease in black Africans with HIV infection: a cross-sectional hospital-based study | Did not report relevant outcomes (cardiovascular outcome) |
| Kauppinen et al. | 2019 | Switching from Tenofovir Disoproxil Fumarate to Tenofovir Alafenamide Significantly Worsens the Lipid Profile in a Real-World Setting | Did not include relevant comparator |
| Keiser et al. | 2005 | Substituting abacavir for hyperlipidemia-associated protease inhibitors in HAART regimens improves fasting lipid profiles, maintains virologic suppression, and simplifies treatment | Did not include relevant comparator |
| Khemla et al. | 2023 | Lipid profiles of people with human immunodeficiency virus with dyslipidemia after switching from efavirenz to dolutegravir | Did not include relevant comparator |
| Kigongo et al. | 2024 | Dyslipidemia among adult people living with HIV on dolutegravir - based antiretroviral therapy at a private tertiary hospital in Kampala, Uganda: burden and determinants | Did not include relevant comparator |
| Kim et al. | 2025 | Safety of tenofovir alafenamide in the context of hyperlipidemia and cardiovascular diseases: a nationwide analysis | Did not include relevant comparator |
| Kim et al. | 2018 | Hypertension Risk with Abacavir Use among HIV-Infected Individuals: A Nationwide Cohort Study | Did not include relevant comparator |
| Kim et al. | 2025 | Tenofovir Disoproxil Fumarate Versus Entecavir: Effects on Lipid Profiles and Cardiovascular Outcomes in People Living With Chronic Hepatitis B | Research question not relevant to review |
| Kim et al. | 2022 | Weight Gain and Lipid Profile Changes in Koreans with Human Immunodeficiency Virus undergoing Integrase Strand Transfer Inhibitor-Based Regimens | Did not report relevant outcomes (metabolic measures) |
| Kingsley et al. | 2008 | Subclinical coronary atherosclerosis, HIV infection and antiretroviral therapy: Multicenter AIDS Cohort Study | Did not report relevant outcomes (cardiovascular outcome) |
| Kintu et al. | 2018 | Incidence and Risk Factors for Overweight and Obesity after Initiation of Antiretroviral Therapy in Dar es Salaam, Tanzania | Did not include relevant comparator |
| Klage et al. | 2013 | Cardiometabolic risk factors among HIV patients on antiretroviral therapy | Did not include relevant comparator |
| Koethe et al. | 2016 | Rising Obesity Prevalence and Weight Gain Among Adults Starting Antiretroviral Therapy in the United States and Canada | Did not include relevant comparator |
| Koethe et al. | 2015 | Body mass index and the risk of incident noncommunicable diseases after starting antiretroviral therapy | Did not include relevant comparator |
| Koppel et al. | 2002 | Hypofibrinolytic state in HIV-1-Infected patients treated with proteasa inhibitor-containing highly active antiretroviral therapy | Did not report relevant outcomes |
| Koppel et al. | 2000 | Serum lipid levels associated with increased risk for cardiovascular disease is associated with highly active antiretroviral therapy (HAART) in HIV-1 infection | Did not report relevant outcomes (metabolic measures) |
| Kousari et al. | 2024 | Weight change with antiretroviral switch from integrase inhibitor or tenofovir alafenamide-based to Doravirine-Based regimens in people with HIV | Did not include relevant comparator |
| Krishnan et al. | 2015 | Changes in metabolic syndrome status after initiation of antiretroviral therapy | Did not include relevant comparator |
| Krishnan et al. | 2012 | Metabolic syndrome before and after initiation of antiretroviral therapy in treatment-naive HIV-infected individuals | Did not include relevant comparator |
| Kristoffersen et al. | 2009 | Changes in biomarkers of cardiovascular risk after a switch to abacavir in HIV-1-infected individuals receiving combination antiretroviral therapy | Did not include relevant comparator |
| Kumar et al. | 2018 | Central obesity &amp; dyslipidemia in HIV patients on antiretroviral therapy | Not an eligible publication type |
| Labhardt et al. | 2017 | Metabolic syndrome in patients on first-line antiretroviral therapy containing zidovudine or tenofovir in rural Lesotho, Southern Africa | Did not include relevant comparator |
| Lafeuillade et al. | 2003 | Comparison of metabolic abnormalities and clinical lipodystrophy 48 weeks after switching from HAART to Trizivir versus continued HAART: the Trizal study | Did not include relevant comparator |
| Lahiri et al. | 2021 | Weight and Body Mass Index Change After Switching to Integrase Inhibitors or Tenofovir Alafenamide Among Women Living with HIV | Did not include relevant comparator |
| Lai et al. | 2003 | Factors associated with accelerated atherosclerosis in HIV-1-infected persons treated with protease inhibitors | Did not include relevant comparator |
| Lai et al. | 2018 | Incidence of cardiovascular diseases in a nationwide HIV/AIDS patient cohort in Taiwan from 2000 to 2014 | Irrelevant age group |
| Lang et al. | 2010 | Impact of individual antiretroviral drugs on the risk of myocardial infarction in human immunodeficiency virus-infected patients: a case-control study nested within the French Hospital Database on HIV ANRS cohort CO4 | Did not include relevant comparator |
| Lartey et al. | 2024 | Hypertension Among Cohort of Persons With Human Immunodeficiency Virus Initiated on a Dolutegravir-Based Antiretroviral Regimen in Ghana | Did not include relevant comparator |
| Liu et al. | 2021 | Gradual increasing dyslipidemia in treatment-naive male patients with human immunodeficiency virus and treated with tenofovir plus lamivudine plus efavirenz for 3 years | Did not include relevant comparator |
| Liu et al. | 2013 | First-line antiretroviral therapy and changes in lipid levels over 3 years among HIV-infected adults in Tanzania | Did not include relevant comparator |
| Llibre et al. | 2006 | Sustained improvement of dyslipidaemia in HAART-treated patients replacing stavudine with tenofovir | Did not include relevant comparator |
| Lowe et al. | 2007 | Stavudine but not didanosine as part of HAART contributes to peripheral lipoatrophy: a substudy from the Antiretroviral Regimen Evaluation Study (ARES) | Did not include relevant comparator |
| Lu et al. | 2011 | Outcomes of switch to atazanavir-containing combination antiretroviral therapy in HIV-1-infected patients with hyperlipidemia | Did not include relevant comparator |
| Lubega et al. | 2021 | Prevalence and factors associated with hypertension among people living with HIV/AIDS on antiretroviral therapy in Uganda | Did not include relevant comparator |
| Mafumhe et al. | 2024 | Comparison of Switching Between Antiretroviral Agents Versus Introducing Lipid-lowering Agents for HAART-induced Dyslipidemia | Not using an eligible study design |
| Magenta et al. | 2011 | Lipid and lipoprotein profile in HIV-infected patients treated with lopinavir/ritonavir as a component of the first combination antiretroviral therapy | Did not include relevant comparator |
| Maggi et al. | 2011 | Cardiovascular risk factors in patients on long-term treatment with nevirapine- or efavirenz-based regimens | Did not include relevant comparator |
| Maggi et al. | 2017 | Cardiovascular risk in advanced naïve HIV-infected patients starting antiretroviral therapy: Comparison of three different regimens - PREVALEAT II cohort | Did not report relevant outcomes |
| Maia Leite et al. | 2010 | Progression to overweight, obesity and associated factors after antiretroviral therapy initiation among Brazilian persons with HIV/AIDS | Did not include relevant comparator |
| Mallon et al. | 2021 | Weight gain before and after switch from TDF to TAF in a U.S. cohort study | Did not include relevant comparator |
| Maman et al. | 2024 | The effect of a treatment switch to integrase Strand transfer inhibitor-based regimens on weight gain and other metabolic syndrome-related conditions | Did not include relevant comparator |
| Manne-Goehler et al. | 2024 | Blood pressure increases are associated with weight gain and not antiretroviral regimen or kidney function: a secondary analysis from the ADVANCE trial in South Africa | Did not report relevant outcomes |
| Manosuthi et al. | 2008 | Effectiveness and metabolic complications after 96 weeks of a generic fixed-dose combination of stavudine, lamivudine, and nevirapine among antiretroviral-naive advanced HIV-infected patients in Thailand: A prospective study | Did not include relevant comparator |
| Marcus et al. | 2016 | Use of abacavir and risk of cardiovascular disease among HIV-infected individuals | Did not report relevant outcomes (cardiovascular outcome) |
| Martin et al. | 2011 | Predictors of limb fat gain in HIV positive patients following a change to tenofovir-emtricitabine or abacavir-lamivudine | Did not include relevant comparator |
| Martin et al. | 2013 | HIV Lipodystrophy in Participants Randomised to Lopinavir/Ritonavir (LPV/r)+2-3 Nucleoside/Nucleotide Reverse Transcriptase Inhibitors (N(t)RTI) or LPV/r plus Raltegravir as Second-Line Antiretroviral Therapy | Did not include relevant comparator |
| Martin et al. | 2004 | Reversibility of lipoatrophy in HIV-infected patients 2 years after switching from a thymidine analogue to abacavir: the MITOX Extension Study | Did not include relevant comparator |
| Martin et al. | 2013 | HIV Lipodystrophy in Participants Randomised to Lopinavir/Ritonavir (LPV/r) +2‚Äì3 Nucleoside/Nucleotide Reverse Transcriptase Inhibitors (N(t)RTI) or LPV/r + Raltegravir as Second-Line Antiretroviral Therapy | Did not include relevant comparator |
| Martinez et al. | 2001 | Risk of lipodystrophy in HIV-1-infected patients treated with protease inhibitors: a prospective cohort study | Did not include relevant comparator |
| Martinez et al. | 2010 | The effects of intermittent, CD4-guided antiretroviral therapy on body composition and metabolic parameters | Did not include relevant comparator |
| Martínez et al. | 2012 | Changes in cardiovascular biomarkers in HIV-infected patients switching from ritonavir-boosted protease inhibitors to raltegravir | Did not include relevant comparator |
| Martínez et al. | 2000 | Impact of switching from human immunodeficiency virus type 1 protease inhibitors to efavirenz in successfully treated adults with lipodystrophy | Did not include relevant comparator |
| Martínez et al. | 2000 | A prospective cohort study on the risk for lipodystrophy in HIV-infected patients treated with protease inhibitor-containing regimens | Did not include relevant comparator |
| Martínez-Sanz et al. | 2021 | Weight changes after antiretroviral therapy initiation in CoRIS (Spain): a prospective multicentre cohort study | Did not include relevant comparator |
| Martínez-Sanz et al. | 2023 | Metabolic-Related Outcomes After Switching From Tenofovir Disoproxil Fumarate to Tenofovir Alafenamide in Adults With Human Immunodeficiency Virus (HIV): A Multicenter Prospective Cohort Study | Did not include relevant comparator |
| Martini et al. | 2022 | Dynamics of Lipid Profile in Antiretroviral-Naive HIV-Infected Patients, Treated with TAF-Based Regimens: A Multicenter Observational Study | Did not include relevant comparator |
| Martini et al. | 2023 | Evaluation of Lipid Profile and Intima Media Thickness in Antiretroviral-Experienced HIV-Infected Patients Treated with Protease Inhibitor-Based Regimens versus Protease Inhibitor-Sparing Regimens | Did not include relevant comparator |
| Marzolini et al. | 2015 | Impact of body weight on virological and immunological responses to efavirenz-containing regimens in HIV-infected, treatment-naive adults | Not an eligible publication type |
| Masiá et al. | 2012 | Cardiovascular risk in human immunodeficiency virus-infected patients in Spain. CoRIS cohort, 2011 | Study not available in English |
| Mathebula et al. | 2020 | The prevalence of selected non-communicable disease risk factors among HIV patients on anti-retroviral therapy in Bushbuckridge sub-district, Mpumalanga province | Did not include relevant comparator |
| Mave et al. | 2016 | Inflammation and Change in Body Weight With Antiretroviral Therapy Initiation in a Multinational Cohort of HIV-Infected Adults | Did not include relevant comparator |
| Mayanja et al. | 2017 | COHORT PROFILE: The Complications of Long-Term Antiretroviral Therapy study in Uganda (CoLTART), a prospective clinical cohort | Did not include relevant comparator |
| McCann et al. | 2021 | Implications of weight gain with newer anti-retrovirals: 10-year predictions of cardiovascular disease and diabetes | Did not include relevant comparator |
| McComsey et al. | 2011 | Peripheral and central fat changes in subjects randomized to abacavir-lamivudine or tenofovir-emtricitabine with atazanavir-ritonavir or efavirenz: ACTG Study A5224s | Did not include relevant comparator |
| Mena et al. | 2019 | Similar plasma lipidomic profile in people living with HIV treated with a darunavir-based or an integrase inhibitor-based antiretroviral therapy | Not conducted in eligible setting |
| Mercier et al. | 2009 | Lipodystrophy and metabolic disorders in HIV-1-infected adults on 4- to 9-year antiretroviral therapy in Senegal: a case-control study | Did not include relevant comparator |
| Milinkovic et al. | 2007 | The impact of reducing stavudine dose versus switching to tenofovir on plasma lipids, body composition and mitochondrial function in HIV-infected patients | Did not include relevant comparator |
| Möbius et al. | 2005 | Switching to atazanavir improves metabolic disorders in antiretroviral-experienced patients with severe hyperlipidemia | Not conducted in eligible setting |
| Monforte et al. | 2013 | Atazanavir is not associated with an increased risk of cardio- or cerebrovascular disease events | Not an eligible publication type |
| Montes et al. | 2024 | Incidence of Diabetes Mellitus and Associated Factors in the Era of Antiretroviral Drugs With a Low Metabolic Toxicity Profile | Did not include relevant comparator |
| Montes et al. | 2005 | Lipid disorders in antiretroviral-naive patients treated with lopinavir/ritonavir-based HAART: frequency, characterization and risk factors | Did not include relevant comparator |
| Moschopoulos et al. | 2023 | Switching from Tenofovir Disoproxil to Tenofovir Alafenamide Fumarate: Impact on Cardiovascular Risk and Lipid Profile in People Living with HIV, an Observational Study | Did not include relevant comparator |
| Moyle et al. | 2015 | A randomized comparative trial of continued abacavir/lamivudine plus efavirenz or replacement with efavirenz/emtricitabine/tenofovir DF in hypercholesterolemic HIV-1 infected individuals | Did not include relevant comparator |
| Msoka et al. | 2018 | Association of HIV-infection, antiretroviral treatment and metabolic syndrome with large artery stiffness: a cross-sectional study | Did not report relevant outcomes |
| Muche Belete et al. | 2021 | Serum Lipid Profiles of Patients Taking Efavirenz-Based Antiretroviral Regimen Compared to Ritonavir-Boosted Atazanavir with an Optimized Background at Zewditu Memorial Hospital, Addis Ababa, Ethiopia | Did not include relevant comparator |
| Muhammad et al. | 2013 | Cardiovascular disease risk factors among HIV-infected Nigerians receiving highly active antiretroviral therapy | Did not report relevant outcomes |
| Mukuna et al. | 2024 | Effect of dolutegravir-based versus efavirenz-based antiretroviral therapy on excessive weight gain in adult treatment-naïve HIV patients at Matsanjeni health center, Eswatini: a retrospective cohort study | Did not include relevant comparator |
| Mulindwa et al. | 2023 | Blood glucose trajectories and incidence of diabetes mellitus in Ugandan people living with HIV initiated on dolutegravir | Did not include relevant comparator |
| Mulindwa et al. | 2023 | Dolutegravir use over 48 weeks is not associated with worsening insulin resistance and pancreatic beta cell function in a cohort of HIV-infected Ugandan adults | Did not include relevant comparator |
| Mullingan et al. | 2005 | Fat distribution in HIV-infected women in the United States: DEXA substudy in the Women's Interagency HIV Study | Not an eligible publication type |
| Mullingan et al. | 2006 | Mixed patterns of changes in central and peripheral fat following initiation of antiretroviral therapy in a randomized trial | Did not include relevant comparator |
| Mulugeta et al. | 2021 | Incidence and Predictors of Hypertension Among HIV Patients Receiving ART at Public Health Facilities, Northwest Ethiopia: A One-Year Multicenter Prospective Follow-Up Study | Did not include relevant comparator |
| Musinguzi et al. | 2021 | Association between obesity and combination antiretroviral therapy (cART) adherence among persons with early-stage HIV infection initiating cART | Did not report relevant outcomes |
| Mutimura et al. | 2007 | Metabolic function and the prevalence of lipodystrophy in a population of HIV-infected African subjects receiving highly active antiretroviral therapy | Did not include relevant comparator |
| Muya et al. | 2019 | Comparative Assessment of the Magnitude of Hyperlipidemia in HIV-Infected Patients Receiving Lopinavir/r- and Atazanavir/r-Based Antiretroviral Drugs | Did not include relevant comparator |
| Narciso et al. | 2001 | Metabolic and morphologic disorders in patients treated with highly active antiretroviral therapy since primary HIV infection | Did not include relevant comparator |
| Neesgaard et al. | 2022 | Associations between integrase strand-transfer inhibitors and cardiovascular disease in people living with HIV: a multicentre prospective study from the RESPOND cohort consortium | Did not include relevant comparator |
| Nguyen et al. | 2008 | Lipodystrophy and weight changes: data from the Swiss HIV Cohort Study, 2000-2006 | Did not include relevant comparator |
| Njelekela et al. | 2017 | Lipodystrophy among HIV-Infected Patients Attending Care and Treatment Clinics in Dar es Salaam | Did not include relevant comparator |
| Nzaramba et al. | 2025 | Low Aspartate Aminotransferase/Alanine Aminotransferase Ratio as an Indicator of Metabolic Syndrome Among HIV Patients on Dolutegravir Therapy in Southwestern Uganda | Did not include relevant comparator |
| O’Nell et al. | 2022 | Assessing risk factors for hypertension in young adults with perinatally acquired HIV infection: A case-control study | Research question not relevant to review |
| Obel et al. | 2009 | Abacavir and risk of myocardial infarction in HIV-infected patients on highly active antiretroviral therapy: a population-based nationwide cohort study | Did not report relevant outcomes (cardiovascular outcome) |
| Ocampo et al. | 2018 | Lipid changes and tolerability in a cohort of adult HIV-infected patients who switched to rilpivirine/emtricitabine/tenofovir due to intolerance to previous combination ART: the PRO-STR study | Did not include relevant comparator |
| Ombajo et al. | 2024 | Change in Blood Pressure, Weight, and Other Cardiovascular Disease Risk Factors After Switch From Protease Inhibitors to Dolutegravir: Post hoc Analysis of the 48-week Randomised Second-line Switch to Dolutegravir Trial | Not an eligible publication type |
| Ombeni et al. | 2016 | Lipid Profile in HIV-Infected Patients Using First-Line Antiretroviral Drugs | Did not include relevant comparator |
| Omech et al. | 2012 | Prevalence of HIV-Associated Metabolic Abnormalities among Patients Taking First-Line Antiretroviral Therapy in Uganda | Did not include relevant comparator |
| Ongolo-Zogo et al. | 2012 | Lipodystrophy and echographic hepatic steatosis in HIV-positive patients under highly active antiretroviral therapy (HAART) in Yaounde (Cameroon) | Study not available in English |
| Osegbe et al. | 2016 | Risk factors and assessment for cardiovascular disease among HIV-positive patients attending a Nigerian tertiary hospital | Did not report relevant outcomes (cardiovascular outcome) |
| Palacios et al. | 2006 | Incidence of and risk factors for insulin resistance in treatment-naive HIV-infected patients 48 weeks after starting highly active antiretroviral therapy | Did not include relevant comparator |
| Palacios et al. | 2006 | Impact of highly active antiretroviral therapy on blood pressure in HIV-infected patients. A prospective study in a cohort of naive patients | Did not include relevant comparator |
| Pangmekeh et al. | 2019 | Association between highly active antiretroviral therapy (HAART) and hypertension in persons living with HIV/AIDS at the Bamenda regional hospital, Cameroon | Did not report relevant outcomes (cardiovascular outcome) |
| Parienti et al. | 2007 | Efavirenz to nevirapine switch in HIV-1-infected patients with dyslipidemia: a randomized, controlled study | Not an eligible publication type |
| Patel et al. | 2012 | Effect of abacavir on acute changes in biomarkers associated with cardiovascular dysfunction | Not an eligible publication type |
| Patroni et al. | 2002 | Effect of highly active antiretroviral therapy (HAART) and hepatitis C Co-infection on hyperlipidemia in HIV-infected patients: a retrospective longitudinal study | Did not include relevant comparator |
| Peck et al. | 2014 | Hypertension, kydney diease, HIV, and antiretroviral therapy among Tanzanian adults a cross-sectional study | Did not report relevant outcomes (cardiovascular outcome) |
| Pefura Yone et al. | 2011 | First-line antiretroviral therapy and dyslipidemia in people living with HIV-1 in Cameroon: a cross-sectional study | Did not report relevant outcomes (metabolic measures) |
| Périard et al. | 1999 | Atherogenic dyslipidemia in HIV-infected individuals treated with protease inhibitors | Did not include relevant comparator |
| Petit et al. | 2004 | Serum adiponectin and metabolic parameters in HIV-1-infected patients after substitution of nevirapine for protease inhibitors | Did not include relevant comparator |
| Petrakis et al. | 2020 | Tenofovir Alafenamide Fumarate Therapy for HIV Treatment: Cardiometabolic and Renal Safety | Did not include relevant comparator |
| Piconi et al. | 2013 | Atherosclerosis is associated with multiple pathogenic mechanisms in HIV-infected antiretroviral-naive or treated individuals | Did not report relevant outcomes (cardiovascular outcome) |
| Pinto Neto et al. | 2013 | Dyslipidemia and fasting glucose impairment among HIV patients three years after the first antiretroviral regimen in a Brazilian AIDS outpatient clinic | Did not include relevant comparator |
| Plum et al. | 2021 | Impact of switch from tenofovir disoproxil fumarate-based regimens to tenofovir alafenamide-based regimens on lipid profile, weight gain and cardiovascular risk score in people living with HIV | Did not include relevant comparator |
| Podzamczer et al. | 2011 | Lipid profiles for nevirapine vs. atazanavir/ritonavir, both combined with tenofovir disoproxil fumarate and emtricitabine over 48 weeks, in treatment-naive HIV-1-infected patients (the ARTEN study) | Did not include relevant comparator |
| Price et al. | 2015 | Changes in the prevalence of lipodystrophy, metabolic syndrome and cardiovascular disease risk in HIV-infected men | Did not include relevant comparator |
| Pujari et al. | 2005 | Lipodystrophy and dyslipidemia among patients taking first-line, World Health Organization-recommended highly active antiretroviral therapy regimens in Western India | Not an eligible publication type |
| Pujari et al. | 2005 | Lipodystrophy and Dyslipidemia Among Patients Taking First-Line, World Health Organization–Recommended Highly Active Antiretroviral Therapy Regimens in Western India | Did not report relevant outcomes (metabolic measures) |
| Quercia et al. | 2015 | Comparative changes of lipid levels in treatment-naive, HIV-1-infected adults treated with dolutegravir vs. efavirenz, raltegravir, and ritonavir-boosted darunavir-based regimens over 48 weeks | Did not include relevant comparator |
| Rakotoambinina et al. | 2001 | Lipodystrophic syndromes and hyperlipidemia in a cohort of HIV-1-infected patients receiving triple combination antiretroviral therapy with a protease inhibitor | Did not include relevant comparator |
| Ranzenigo et al. | 2024 | Cabotegravir and rilpivirine reduce the risk of developing metabolic syndrome | Did not include relevant comparator |
| Rebeiro et al. | 2023 | Incidence of cardiometabolic outcomes among people living with HIV-1 initiated on integrase strand transfer inhibitor versus non-integrase strand transfer inhibitor antiretroviral therapies: a retrospective analysis of insurance claims in the United States | Did not include relevant comparator |
| Ribera et al. | 2013 | Impact of switching from zidovudine/lamivudine to tenofovir/emtricitabine on lipoatrophy: the RECOMB study | Did not include relevant comparator |
| Ribera et al. | 2008 | Improvements in subcutaneous fat, lipid profile, and parameters of mitochondrial toxicity in patients with peripheral lipoatrophy when stavudine is switched to tenofovir (LIPOTEST study) | Did not include relevant comparator |
| Rimland et al. | 2006 | Antiretroviral therapy in HIV-positive women is associated with increased apolipoproteins and total cholesterol | Did not report relevant outcomes (metabolic measures) |
| Rodríguez-Arbolí et al. | 2017 | Incidence and risk factors for hypertension among HIV patients in rural Tanzania - A prospective cohort study | Did not include relevant comparator |
| Rodríguez-Gallego et al. | 2018 | Circulating metabolomic profile can predict dyslipidemia in HIV patients undergoing antiretroviral therapy | Did not include relevant comparator |
| Romo et al. | 2024 | Dolutegravir-containing antiretroviral therapy and incident hypertension: findings from a prospective cohort in Kenya, Nigeria, Tanzania and Uganda | Did not report relevant outcomes |
| Rotea-Salvo et al. | 2023 | Lipid profile changes associated with antiretroviral therapies in a real-world cohort | Did not include relevant comparator |
| Rugera et al. | 2025 | Association of obesity with hyperuricaemia among HIV-positive patients on antiretroviral therapy in South-Western Uganda | Research question not relevant to review |
| Ruiz et al. | 2001 | Antiretroviral treatment simplification with nevirapine in protease inhibitor-experienced patients with hiv-associated lipodystrophy: 1-year prospective follow-up of a multicenter, randomized, controlled study | Did not include relevant comparator |
| Ryom et al. | 2018 | Cardiovascular disease and use of contemporary protease inhibitors: the D:A:D international prospective multicohort study | Did not include relevant comparator |
| Sahagun et al. | 2022 | Associations Between Central Obesity and Lifelong Antiviral Therapy in Adults Living With HIV Acquired From Early Childhood | Did not include relevant comparator |
| Salami et al. | 2009 | Serum lipids and glucose abnormalities in HIV/AIDS patients on antiretroviral therapies | Did not include relevant comparator |
| Santiprabhob et al. | 2024 | Adipocytokine dysregulation, abnormal glucose metabolism, and lipodystrophy in HIV-infected adolescents receiving protease inhibitors (vol 136, 155145, 2020) | Not an eligible publication type |
| Sarfo et al. | 2020 | Duration of antiretroviral therapy among people living with HIV and incidence of hypertension in Ghana | Did not include relevant comparator |
| Sarfo et al. | 2019 | Characteristics of hypertension among people living with HIV in Ghana: Impact of new hypertension guideline | Did not report relevant outcomes (cardiovascular outcome) |
| Saumoy et al. | 2011 | Low-density lipoprotein size and lipoprotein-associated phospholipase A2 in HIV-infected patients switching to abacavir or tenofovir | Did not include relevant comparator |
| Saumoy et al. | 2022 | Atherogenicity of low-density lipoproteins after switching from a protease inhibitor to dolutegravir: a substudy of the NEAT022 study | Did not include relevant comparator |
| Schafer et al. | 2022 | Weight changes in patients with sustained viral suppression switching tenofovir disoproxil fumarate to tenofovir alafenamide | Did not include relevant comparator |
| Segarra-Newnham, M. | 2002 | Hyperlipidemia in HIV-positive patients receiving antiretrovirals | Did not include relevant comparator |
| Segerer et al. | 1999 | Hyperlipidemia under treatment with proteinase inhibitors | Did not include relevant comparator |
| Sempere et al. | 2023 | Incidence of Hypertension and Blood Pressure Changes in Persons With Human Immunodeficiency Virus at High Risk for Cardiovascular Disease Switching From Boosted Protease Inhibitors to Dolutegravir: A Post-hoc Analysis of the 96-week Randomised NEAT-022 Trial | Did not include relevant comparator |
| Sension et al. | 2009 | Improvement in lipid profiles in antiretroviral-experienced HIV-positive patients with hyperlipidemia after a switch to unboosted atazanavir | Did not include relevant comparator |
| Shaffer et al. | 2014 | Cardiovascular disease risk factors in HIV-infected women after initiation of lopinavir/ritonavir- and nevirapine-based antiretroviral therapy in Sub-Saharan Africa: A5208 (OCTANE) | Did not include relevant comparator |
| Shamu et al. | 2024 | Body weight and blood pressure changes on dolutegravir-, efavirenz- or atazanavir-based antiretroviral therapy in Zimbabwe: a longitudinal study | Did not include relevant comparator |
| Sharma et al. | 2008 | Adverse effects of antiretroviral treatment | Did not include relevant comparator |
| Shidhaye et al. | 2023 | Metabolic syndrome in people living with antiretroviral therapy: A cross-sectional investigation from Pune, India | Unable to retrieve full text |
| Shikuma et al. | 2004 | Changes in weight and lean body mass during highliy active ART | Did not report relevant outcomes (metabolic measures) |
| Shlay et al. | 2005 | Body composition and metabolic changes in antiretroviral-naive patients randomized to didanosine and stavudine vs. abacavir and lamivudine | Did not include relevant comparator |
| Siddiqui et al. | 2022 | Risk Factors for Incident Hypertension Within 1 Year of Initiating Antiretroviral Therapy Among People with HIV | Did not include relevant comparator |
| Sinha et al. | 2022 | An Observational Study on Metabolic Derangements in People Living with HIV | Did not report relevant outcomes (metabolic measures) |
| Smith et al. | 2004 | Cardiovascular disease risk factors and antiretroviral therapy in an HIV-positive UK population | Did not report relevant outcomes |
| Smith et al. | 2004 | Original Research Cardiovascular disease risk factors and antiretroviral therapy in an HIV-positive UK population | Did not report relevant outcomes |
| Soares et al. | 2015 | Discordance between body mass index and anthropometric measurements among HIV-1-infected patients on antiretroviral therapy and with lipoatrophy/lipohypertrophy syndrome | Did not report relevant outcomes (metabolic measures) |
| Steffen et al. | 2023 | Blood pressure changes during tenofovir-based antiretroviral therapy among people living with HIV in Lilongwe, Malawi: results from the prospective LighTen Cohort Study | Did not include relevant comparator |
| Steffen et al. | 2024 | Blood pressure and weight changes and incidence of arterial hypertension 3 years after switching to Dolutegravir in treatment-experienced HIV-infected patients in Lilongwe, Malawi | Not an eligible publication type |
| Stein et al. | 2008 | Lipoprotein Changes in HIV-Infected Antiretroviral-Naive Individuals after Starting Antiretroviral Therapy: ACTG Study A5152s Stein: Lipoprotein Changes on Antiretroviral Therapy | Did not include relevant comparator |
| Stein et al. | 2015 | A prospective, randomized clinical trial of antiretroviral therapies on carotid wall thickness | Did not report relevant outcomes |
| Sterne et al. | 2007 | HAART and the heart: changes in coronary risk factors and implications for coronary risk in men starting antiretroviral therapy | Did not include relevant comparator |
| Su et al. | 2012 | The influence of long-term nucleotide reverse transcriptase inhibitors on lipids metabolism in HIV/AIDS patients | Unable to retrieve full text |
| Sun et al. | 2020 | Evolution of blood lipids and risk factors of dyslipidemia among people living with human immunodeficiency virus who had received first-line antiretroviral regimens for 3 years in Shenzhen | Did not include relevant comparator |
| Sun et al. | 2022 | Higher Risk of Dyslipidemia With Coformulated Elvitegravir, Cobicistat, Emtricitabine, and Tenofovir Alafenamide than Efavirenz, Lamivudine, and Tenofovir Disoproxil Fumarate Among Antiretroviral-Naive People Living With HIV in China | Did not include relevant comparator |
| Surial et al. | 2023 | Impact of Integrase Inhibitors on Cardiovascular Disease Events in People With Human Immunodeficiency Virus Starting Antiretroviral Therapy | Did not include relevant comparator |
| Surial et al. | 2021 | Weight and Metabolic Changes After Switching From Tenofovir Disoproxil Fumarate to Tenofovir Alafenamide in People Living With HIV : A Cohort Study | Did not include relevant comparator |
| Taramasso et al. | 2018 | Improvement of lipid profile after switching from efavirenz or ritonavir-boosted protease inhibitors to rilpivirine or once-daily integrase inhibitors: results from a large observational cohort study (SCOLTA) | Did not include relevant comparator |
| Taramasso et al. | 2019 | Lipid profile changings after switching from rilpivirine/tenofovir disoproxil fumarate/emtricitabine to rilpivirine/tenofovir alafenamide/emtricitabine: Different effects in patients with or without baseline hypercholesterolemia | Did not include relevant comparator |
| Tebas et al. | 2014 | Lipid levels and changes in body fat distribution in treatment-naive, HIV-1-Infected adults treated with rilpivirine or Efavirenz for 96 weeks in the ECHO and THRIVE trials | Did not include relevant comparator |
| Tebas et al. | 2007 | Switching to a protease inhibitor-containing, nucleoside-sparing regimen (lopinavir/ritonavir plus efavirenz) increases limb fat but raises serum lipid levels: results of a prospective randomized trial (AIDS clinical trial group 5125s) | Did not include relevant comparator |
| Temesgen et al. | 2019 | Hypertension in HIV-Infected Patients Receiving Antiretroviral Therapy in Northeast Ethiopia | Did not assess relevant exposures |
| Thamrongwonglert et al. | 2016 | Improvement of lipid profiles when switching from efavirenz to rilpivirine in HIV-infected patients with dyslipidemia | Did not include relevant comparator |
| The RESPOND Study Group | 2021 | Incidence of dyslipidemia in people with HIV who are treated with integrase inhibitors versus other antiretroviral agents | Unable to retrieve full text |
| Thiébaut et al. | 2001 | Incidence and risk factors of severe hypertriglyceridaemia in the era of highly active antiretroviral therapy: the Aquitaine Cohort, France, 1996-99 | Did not include relevant comparator |
| Tiarukkitsagul et al. | 2021 | Assessment of atherosclerotic cardiovascular disease risks between people living with HIV receiving first-line and second-line antiretroviral therapy in a resource-limited setting | Did not include relevant comparator |
| Tieosapjaroen et al. | 2023 | Weight Change When Initiating, Switching to, and Discontinuing Integrase Strand Transfer Inhibitors in People Living with HIV | Did not include relevant comparator |
| Tiozzo et al. | 2015 | A cross-sectional assessment of metabolic syndrome in HIV-infected people of low socioeconomic status receiving antiretroviral therapy | Did not include relevant comparator |
| Trifirò et al. | 2023 | Hypertension in people living with HIV on combined antiretroviral therapy in rural Tanzania | Did not assess relevant exposures |
| Tsekes et al. | 2002 | Body composition changes in protease inhibitor-naive HIV-infected patients treated with two nucleoside reverse transcriptase inhibitors | Did not include relevant comparator |
| Tsiodras et al. | 2000 | Effects of protease inhibitors on hyperglycemia, hyperlipidemia, and lipodystrophy: a 5-year cohort study | Did not include relevant comparator |
| Ucciferri et al. | 2013 | Improved metabolic profile after switch to darunavir/ritonavir in HIV positive patients previously on protease inhibitor therapy | Did not include relevant comparator |
| Valantin et al. | 2012 | Body fat distribution in HIV-infected patients treated for 96 weeks with darunavir/ritonavir monotherapy versus darunavir/ritonavir plus nucleoside reverse transcriptase inhibitors: the MONOI-ANRS136 substudy | Did not include relevant comparator |
| van der Valk et al. | 2001 | Lipodystrophy in HIV-1-positive patients is associated with insulin resistance in multiple metabolic pathways | Did not include relevant comparator |
| van der Valk et al. | 2001 | Increased risk of lipodystrophy when nucleoside analogue reverse transcriptase inhibitors are included with protease inhibitors in the treatment of HIV-1 infection | Did not include relevant comparator |
| van der Valk et al. | 2001 | Nevirapine-containing antiretroviral therapy in HIV-1 infected patients results in an anti-atherogenic lipid profile | Did not include relevant comparator |
| van Griensven et al. | 2007 | High prevalence of lipoatrophy among patients on stavudine-containing first-line antiretroviral therapy regimens in Rwanda | Did not include relevant comparator |
| van Griensven et al. | 2009 | Weight evolution in HIV-1 infected women in Rwanda after stavudine substitution due to lipoatrophy: comparison of zidovudine with tenofovir/abacavir | Did not include relevant comparator |
| van Vonderen et al. | 2009 | Baseline lipid levels rather than the presence of reported body shape changes determine the degree of improvement in lipid levels after switching to atazanavir | Did not include relevant comparator |
| van Vonderen et al. | 2009 | Carotid intima-media thickness and arterial stiffness in HIV-infected patients: the role of HIV, antiretroviral therapy, and lipodystrophy | Did not report relevant outcomes |
| van Wyk et al. | 2021 | Brief Report: Improvement in Metabolic Health Parameters at Week 48 After Switching From a Tenofovir Alafenamide-Based 3- or 4-Drug Regimen to the 2-Drug Regimen of Dolutegravir/Lamivudine: The TANGO Study | Not an eligible publication type |
| Varriano et al. | 2021 | Cardiovascular Events in an Inner-City HIV Clinic and Relationship to Abacavir Versus Tenofovir Disoproxil Fumarate-Containing Antiretroviral Regimens | Unable to retrieve full text |
| Verburgh et al. | 2022 | One in 10 Virally Suppressed Persons With HIV in The Netherlands Experiences Weight Gain After Switching to Tenofovir Alafenamide and/or Integrase Strand Transfer Inhibitor | Did not include relevant comparator |
| Vigouroux et al. | 1999 | Diabetes, insulin resistance and dyslipidaemia in lipodystrophic HIV-infected patients on highly active antiretroviral therapy (HAART) | Did not include relevant comparator |
| Vos et al. | 2018 | Lipid levels, insulin resistance and cardiovascular risk over 96¬weeks of antiretroviral therapy: a randomised controlled trial comparing low-dose stavudine and tenofovir | Did not include relevant comparator |
| Vos et al. | 2019 | Cardiovascular disease risk in an urban African population: a cross-sectional analysis on the role of HIV and antiretroviral treatment | Did not report relevant outcomes (cardiovascular outcome) |
| Vrouenraets et al. | 2011 | Randomized comparison of metabolic and renal effects of saquinavir/r or atazanavir/r plus tenofovir/emtricitabine in treatment-naive HIV-1-infected patients | Did not include relevant comparator |
| Walli et al. | 2001 | Improvement of HAART-associated insulin resistance and dyslipidemia after replacement of protease inhibitors with abacavir | Unable to retrieve full text |
| Walli et al. | 1998 | Treatment with protease inhibitors associated with peripheral insulin resistance and impaired oral glucose tolerance in HIV-1-infected patients | Did not report relevant outcomes (metabolic measures) |
| Walmsley et al. | 2008 | A prospective study of body fat redistribution, lipid, and glucose parameters in HIV-infected patients initiating combination antiretroviral therapy | Did not include relevant comparator |
| Wand et al. | 2007 | Metabolic syndrome, cardiovascular disease and type 2 diabetes mellitus after initiation of antiretroviral therapy in HIV infection | Did not include relevant comparator |
| Wang et al. | 2024 | Efficacy and effect on lipid profiles of switching to ainuovirine-based regimen versus continuing efavirenz-based regimen in people with HIV-1: 24-week results from a real-world, retrospective, multi-center cohort study | Did not include relevant comparator |
| Wang et al. | 2024 | Changes in blood lipid levels and influencing factors among treatment-naive adult male HIV/AIDS patients following BIC/FTC/TAF vs. 3TC+EFV+TDF | Did not include relevant comparator |
| Wang et al. | 2024 | The risk of dyslipidemia on PLHIV associated with different antiretroviral regimens in Huzhou | Did not include relevant comparator |
| Wangsomboonsiri et al. | 2010 | Association between HLA-B*4001 and lipodystrophy among HIV-infected patients from Thailand who received a stavudine-containing antiretroviral regimen | Did not include relevant comparator |
| Waritu et al. | 2024 | Serum Lipid Profiles, Blood Glucose, and High-Sensitivity C-Reactive Protein Levels Among People Living with HIV Taking Dolutegravir and Ritonavir-Boosted Atazanavir-Based Antiretroviral Therapy at Jimma University Medical Center, Southwest Ethiopia, 2021 | Did not include relevant comparator |
| Waters et al. | 2023 | Limited Weight Impact After Switching From Boosted Protease Inhibitors to Dolutegravir in Persons With Human Immunodeficiency Virus With High Cardiovascular Risk: A Post Hoc Analysis of the 96-Week NEAT-022 Randomized Trial | Did not include relevant comparator |
| Weisser et al. | 2025 | Body weight changes in people with HIV starting dolutegravir versus efavirenz-based regimens in a large cohort in rural Tanzania | Did not include relevant comparator |
| Williams et al. | 2009 | Improvement in lipid profiles over 6 years of follow-up in adults with AIDS and immune reconstitution | Did not include relevant comparator |
| Wilson et al. | 2009 | Effects of HIV status and antiretroviral therapy on blood pressure | Did not report relevant outcomes (cardiovascular measures) |
| Wu et al. | 2024 | Metabolic Outcomes of Changing From Rilpivirine/Tenofovir Disoproxil Fumarate/Emtricitabine to Rilpivirine/Tenofovir Alafenamide/Emtricitabine: A Longitudinal Study | Did not include relevant comparator |
| Xu et al. | 2021 | General and abdominal obesity and incident hypertension among people living with HIV on antiretroviral therapy | Did not include relevant comparator |
| Xu et al. | 2024 | Interaction between antiretroviral therapy regimens and body mass index on triglyceride levels in people living with HIV: a cross-sectional and longitudinal study | Did not include relevant comparator |
| Yakut, A. | 2024 | Retrospective cohort evaluation study in terms of cardiovascular and metabolic diseases in chronic hepatitis B patients | Research question not relevant to review |
| Yazie | 2020 | Dyslipidemia and Associated Factors in Tenofovir Disoproxil Fumarate-Based Regimen Among Human Immunodeficiency Virus-Infected Ethiopian Patients: A Hospital-Based Observational Prospective Cohort Study | Did not include relevant comparator |
| Yong et al. | 2013 | Inflammatory biomarker changes and their correlation with Framingham cardiovascular risk and lipid changes in antiretroviral-naive HIV-infected patients treated for 144 weeks with abacavir/lamivudine/atazanavir with or without ritonavir in ARIES | Did not include relevant comparator |
| Yong et al. | 2005 | Body fat changes among antiretroviral-naive patients on PI- and NNRTI-based HAART in the Swiss HIV cohort study | Did not include relevant comparator |
| Yong et al. | 2005 | Lipid profiles for antiretroviral-naive patients starting PI- and NNRTI-based therapy in the Swiss HIV cohort study | Did not include relevant comparator |
| Yong et al. | 2015 | Effects of raltegravir combined with tenofovir/emtricitabine on body shape, bone density, and lipids in African-Americans initiating HIV therapy | Did not include relevant comparator |
| Zhang et al. | 2024 | Impacts of ainuovirine-based and efavirenz-based antiretroviral therapies on the lipid profile of HIV/AIDS patients in southern China: a real world-study | Did not report relevant outcomes (metabolic measures) |
| Zhou et al. | 2014 | To evaluate the changes in body composition in male human immunodeficiency virus-related lipodystrophy after different treatment regimens by dual-energy X-ray absorptiometry | Unable to retrieve full text |
